# Application of allogeneic adult mesenchymal stem cells in the treatment of venous ulcers: A phase I/II randomized controlled trial protocol

**DOI:** 10.1371/journal.pone.0323173

**Published:** 2025-05-15

**Authors:** Víctor J. Costela-Ruiz, Encarnación González-Vigil, Olga Espinosa-Ibáñez, Rosario Mata Alcázar – Caballero, Lucía Melguizo-Rodríguez, Olga Fernández-López, Salvador Arias-Santiago

**Affiliations:** 1 Biomedical Group (BIO277), Department of Nursing, Faculty of Health Sciences, University of Granada, Granada, Spain; 2 Instituto Investigación Biosanitaria, ibs. Granada, Spain; 3 Andalusian Health Service, Granada Metropolitan Health District, Primary Care Unit of Atarfe (Granada), Granada, Spain; 4 Tissue Engineering and Cell Production Unit, Hospital Universitario Virgen de las Nieves, Granada, Spain; 5 Andalusian Network for the Design and Translation of Advanced Therapies, Junta de Andalucía, Seville, Spain; 6 Dermatology Department, Hospital Universitario Virgen de las Nieves, Granada. Spain; 7 Dermatology Department, School of Medicine, University of Granada, Granada, Spain; Saveetha Institute of Medical and Technical Sciences: SIMATS Deemed University, INDIA

## Abstract

**Objective:**

To evaluate the feasibility, safety and efficacy of the cutaneous application of Bioengineered Artificial Mesenchymal Sheet (BAMS) in venous leg ulcers (VLUs) versus conventional treatment.

**Methods:**

This protocol is based on the design of a Phase I/II, multicenter, randomized, controlled, open-label clinical trial investigating the application of a biological dressing supplemented with mesenchymal stem cells (NCT05962931). The clinical trial is being conducted in 2 primary care units within the Granada Metropolitan Health District. A total of 20 patients with VLUs are being randomized (1:1) into 2 intervention arms: a control group and a treatment group. The intervention in the treatment group consists of the local application of 4 doses of BAMS, administered once per week, while the control group receives conventional therapy. Feasibility will be assessed based on the ability to complete the administration of 4 doses in at least 80% of the patients in the treatment group. Safety will be evaluated by analyzing the incidence of adverse effects and serious adverse effects. Efficacy will be assessed in terms of the percentage of wound closure (measured by wound area reduction), macroscopic assessment of the lesion (visual macroscopic analysis and RESVECH 2.0 scale), analysis of growth factors and inflammatory cytokines (ELISA test), pain levels (VAS scale) and quality of life (CIVIQ 20).

**Results:**

If confirmed, BAMS-based therapy may provide an effective treatment for VLUs, potentially reducing wound closure time and associated complications. This therapy could significantly enhance patients’ quality of life due to the regenerative and analgesic properties of the biological dressing.

**Discussion:**

Given the biological activity of mesenchymal stem cells, an accelerated healing effect is expected in the treatment group. This could lead to shorter healing times for chronic wounds, resulting in significant benefits for patients, healthcare professionals, and overall healthcare costs.

**Trial registration:**

NCT05962931.

## Introduction

Wound healing is a complex process that begins with injury and culminates in successful wound closure. It typically progresses through 4 overlapping phases: hemostasis/coagulation, inflammation, proliferation, and maturation/remodeling. Cytokines and growth factors play a crucial role in regulating this process. However, in chronic wounds, the healing process is often disrupted, with the wound failing to progress beyond the inflammatory phase [1]. The wound healing process, though a natural and sequential biological mechanism, can be influenced by various factors, including age, obesity, stress, underlying diseases, lifestyle habits, infections, and trauma, among others. In this sense, there are certain conditions in which the healing process stalled in the inflammatory phase often due to chronic pathologies [2]. As a result, many wounds considered as chronic fail to achieve complete healing, which can lead to complications such as impairment in quality of life, amputations, and even mortality [3]. Thus, a chronic wound is defined as one that does not progress through a normal, orderly and timely repair sequence, or in which the repair process fails to reestablish anatomical and functional integrity after 3 months [4].

In 2014, the cost of wound treatment for Medicare beneficiaries in the United States was estimated to range between $28 to $96.8 billion [5]. In Spain, the annual cost of treating wounds such as pressure ulcers is estimated to be between 461 and 602 million euros. Additionally, between 1.5 and 3% of the total budget of the National Health Systems across the European Union, is allocated to the direct and indirect costs associated with ulcers of any etiology [6–9]. A 2012 study in Germany reported a prevalence of chronic non-healing wounds of 1% to 2% in the general population [10]. Furthermore, in the United States, the annual market for wound care products is estimated to reach $15 to $22 billion by 2024 [11]. Among chronic wounds, diabetic ulcers are the most important and involve the most complications, preceding 85% of all amputations [12].

Venous lower limb ulcers are a type of chronic skin injury caused by elevated venous pressure in the legs [13]. The prevalence of active venous ulcers (VLUs) in the lower limbs ranges from 1.5 to 3 cases per 1000 people with the prevalence increasing with age to approximately 20 per 1000 people by the age of 80 years. Thus, various studies have shown a combined prevalence of 0.32% and a combined incidence of 0.17% of this type of wound. On the other hand, the prevalence of VLUs varies depending on the healthcare setting. In primary care, a global wound prevalence of 0.22% has been reported, with a prevalence of 3.58% in home care patients and 6.56% in nursing homes. These lesions cause significant morbidity and significant healthcare expenditure [14–16]. Thus, the selection of primary care settings in this study is so appropriate and ensures real-world applicability, which enhances the external validity of the finding [17].

The pathophysiology of these injuries is multifactorial. Thus, greatly reduced levels of TGF-β expression have been reported in biopsies of chronic venous – type ulcers [18]. Other histological studies in VLUs have shown the presence of a characteristic hyperproliferative epidermal rim at the wound´s perimeter, alongside a base with an exudate laden with necrotic-type debris. In areas where granulation tissue should be present, vessels surrounded by fibrin, limited neovascularization and a lack of myofibroblasts have been observed, possibly related to the sustained inflammatory infiltrate typical of this type of wound [19]. Regarding healing time, it is clear that this type of wounds do not reduce its size with standard treatment during the first 4 weeks of treatment, with only a small percentage showing a reduction in wound area after this period [20]. In this regard, previous studies indicate that Mesenchymal Stem Cell-based dressings require repeated administration to maximise the therapeutic effects. Therefore, in our study, a weekly dressing schedule for 4 weeks was proposed [21].

The regeneration and healing of complex chronic wounds involves a wide array of therapeutic methodologies [22,23]. However, healing such lesions present a major challenge due to the clinical variability in their therapeutic approaches, as well as the complications related to the delay of all the processes that should occur in a normal healing [24,25]. In this context, pain represents a very complex and widespread symptom that is often associated with difficult-to-heal lower limb ulcers and is often not adequately addressed due to its complexity and the long evolution of treatment [26].

Standard treatments for venous ulcers, including compression therapy, advanced wound dressings, and surgical interventions, are designed to promote wound closure and address the underlying venous pathology [27]. However, these approaches often fail in patients with complex or refractory ulcers, with recurrence rates reaching 70% over 5 years [28]. Emerging regenerative therapies, such as platelet-rich plasma (PRP), growth factor applications, and cellular-based interventions, have shown potential in enhancing wound healing by modulating inflammation, promoting angiogenesis, and improving extracellular matrix remodeling [29]. In this sense, tissue engineering has provided effective and safe alternatives to traditional approaches to wound healing and tissue regeneration. One of the earliest innovations in tissue engineering was the development of artificial skin designed to address certain skin-related pathologies. Over the last decades, several synthetic or bioengineered substitutes have been developed, with the primary goal of being applied to skin lesions. These substitutes not only serve as a protective barrier against microorganisms but also help reduce wound pain and promote healing through tissue regeneration [30–38].

Among tissue regeneration treatments based on cell therapy, mesenchymal stem cells (MSCs) have been a true revolution due to their unique combination of immunomodulatory, anti-inflammatory, and pro-regenerative properties, which target multiple pathological mechanisms underlying chronic wounds [39,40]. MSC are a non-hematopoietic population derived from the mesoderm and are clonogenic in nature. These are cells derived from different tissues such as adipose tissue, bone marrow, dental pulp, placenta, and umbilical cord, among others [41]. One of the key features of this cell line is its pluripotency, which refers to its capacity to differentiate into different lineages, mainly adipocytes, osteocytes and chondrocytes. In addition, these cells have an important immunomodulatory activity and play crucial roles in several diseases related to inflammatory processes. Their involvement in inflammation and tissue damage makes them particularly relevant in the context of wound healing [41–44].

The use of MSCs represents a significant innovation in wound therapy, offering multifaceted mechanisms compared to existing treatments. Conventional therapies such as compression therapy, advanced dressings, and topical growth factor applications primarily focus on creating a favorable environment for healing, whereas MSCs directly address the cellular and molecular disruptions underlying impaired wound healing [27]. For instance, unlike growth factors that stimulate specific processes like angiogenesis, MSCs release a diverse array of bioactive molecules, including anti-inflammatory cytokines and proangiogenic factors, enabling modulation of multiple stages of tissue repair [39]. Similarly, unlike platelet-rich plasma (PRP), which relies on autologous components and may exhibit variable efficacy, MSCs can be applied allogeneically with consistent therapeutic effects, broadening their clinical applicability [40]. Furthermore, MSCs interact directly with immune cells such as macrophages and T lymphocytes, promoting an anti-inflammatory state that supports tissue regeneration while minimizing fibrosis, a common limitation of current therapies [45]. Preclinical and early-phase clinical studies have demonstrated that MSCs can enhance vascularization, modulate excessive inflammation, and support tissue regeneration in chronic wounds, offering a multifaceted therapeutic approach superior to single-target modalities [46].

Scientific evidence has shown the usefulness of allogeneic adult mesenchymal stem cells from adipose tissue (ATMSCs) in the healing of different chronic wounds such as diabetic foot, ulcers and burns, among others. ATMSCs exhibit antalgic properties and accelerate healing by secreting factors that promote angiogenesis [vascular endothelial growth factor (VEGF)] and stimulate the migration of endogenous progenitor cells to the wound bed, such as endothelial cells. Additionally, ATMSCs contribute to the production of extracellular matrix and possess the capacity to differentiate into certain cell groups such as fibroblasts, myofibroblasts and keratinocytes. These cells also demonstrate immunosuppressive effects that confer them anti-inflammatory properties [47–62]. Regarding this last property, ATMSCs can modulate the immune response in inflammatory microenvironments, a mechanism known as MSC polarization [63]. Thus, in the presence of high levels of cytokines and/or proinflammatory factors such as IL-6, interferon gamma (IFN-γ) or tumor necrosis factor alpha (TNF-α), MSCs produce anti-inflammatory and regenerative factors such as transforming growth factor beta (TGF-β) or hepatocyte growth factor (HGF) [64–67].

In this context, the use of dressings with a biological-cellular component has been a real revolution in the treatment of chronic skin lesions. It is important to note that traditional dressings that are routinely used for the treatment of these type of wounds are mainly designed for dry wounds without exudate. Despite their ability to absorb exudate and drain the wound, they require constant changes to avoid maceration and adhesion to the wound surface; which is a painful and tedious process for the patient [68]. Compared to traditional type dressings, other dressings have emerged in recent years that improve the conditions of wound surface wettability, exudate absorption and improved autolytic wound debridement. Thus, biological dressings, specially those based on hydrogels, moisture management and skin-targeting substances, present a wide potential as targeted delivery systems for drugs, antibiotics, nanoparticles, growth factors, regulatory peptides, and even cellular components [69–75].

Regarding the use of biological preparations based on fibrin - hyaluronic acid in tissue regeneration, several studies have shown their stability, effectiveness and bioactive properties. In an *in vitro* study with umbilical cord endothelial cells, Mohandas et al. (2015) demonstrated the positive effect on cell regeneration of a biological dressing made with chitosan, hyaluronic acid and VEGF-loaded nanoparticles. The results showed an increase in angiogenesis in this cell type with the use of this preparation [76]. On the other hand, a randomized clinical trial used a dressing combining platelet-rich fibrin and hyaluronic acid for the treatment of diabetic ulcers. This study showed that the experimental group treated with the platelet-rich fibrin and hyaluronic acid dressing had increased angiogenesis and decreased pathways mediating inflammation [77]. Hinsenkamp et al. (2022), used soft tissue implants prepared with hyaluronic acid alone or in combination with fibrin. The results showed that the use of this implant favoured the biological remodelling process, promoting neovascularisation and the infiltration of cells and extracellular matrix [78].

Based on the variability of treatments and the ineffectiveness of most traditional therapies related to the treatment of VLUs, and considering the regenerative potential of MSCs, the use of a dressing with a cellular component is proposed for the treatment of this type of lesions. So far there are no ongoing trials using the construct. Thus, this phase I/II clinical trial aims to evaluate the feasibility, therapeutic efficacy/effectiveness and safety of the local administration of a Bioengineered Artificial Mesenchymal Sheet (BAMS) in the treatment of VLUs. A phase I/II clinical trial design allows for the simultaneous assessment of safety and preliminary efficacy, which is crucial for novel biologic therapies [79]. Outcome measures including wound closure rate, macroscopic assessment, biomarker analysis, pain and quality of life have been selected following established guidelines for evaluating wound healing interventions [80].

## Methods

### Aims

The aim of this clinical trial is to evaluate the feasibility and the safety of the cutaneous application of the BAMS in VLUs versus conventional treatment. As a primary research approach, it is proposed whether therapy based on the BAMS could be effective in the management of VLUs and the complications associated with these wounds.

As secondary objectives, the following are proposed

- To determine the effect of BAMS on wound closure time in comparison with the control group.
- To evaluate the effect of the experimental treatment in comparison with the control group, on the scar evolution of the lesion in terms of depth, dimension, type of borders, tissue and exudate.
- To analyze the effect of experimental treatment on the concentration of cytokines and growth factors closely related to wound healing (IL-6, IL-4, TGF-β1 and IL-10) in lesion exudate at day 0, + 7, + 14, + 21, + 28, compared to the control group.
- To evaluate the evolution of pain derived from the presence of VLUs in the experimental group, in comparison with the control group.
- To determine the perceived quality of life after treatment with BAMS, compared to the control group.

### Design

This study is a phase I/II 2 arms clinical trial multicenter, randomized 1:1, controlled, open – label, proof of concept (NCT05962931). Each arm will consist of 10 patients who will receive weekly treatment for 4 weeks: the experimental group will receive the local application of BAMS (S1 File) while the control group will receive conventional treatment (S2 File**).**

### Setting of the study

The clinical trial is currently in the patient recruitment phase. Recruitment is being carried out in the 2 participating primary care centers (Atarfe Primary Care Unit and Alfacar Primary Care Unit) located in Granada, Andalusia, Spain. The patients are being randomized by the clinical trial sponsor (Andalusian Network for the Design and Translation of Advanced Therapies – And&tAT).

### Sample size and recruitment

20 patients with VLUs, will be included and randomized a 1:1 ratio into 2 intervention arms: the experimental group and the control group (10 patients in each group).

The 10 patients in the experimental arm will receive 4 doses of the investigational drug (BAMS) applied to the wound bed (S1 File), while the 10 patients in the control group will be treated with conventional therapy (S2 File).

This is a phase I/II clinical trial, proof of concept, of which there are no previous studies, so no mathematical calculation of the sample size has been made. The sample size of 20 individuals was selected considering that this study has a pilot design, the main objective of which is to assess the feasibility, safety and preliminary efficacy of the proposed intervention. Pilot trials are intended to obtain initial estimates of key parameters, such as recruitment rates, protocol adherence and variability of outcome measures, which will be useful for calculating the appropriate sample size in subsequent larger studies [81]. In addition, this sample size is sufficient to identify potential logistical problems and provide information on treatment safety. Finally, given the exploratory nature of this study and resource constraints, a smaller sample size is ethical and appropriate at this early stage of research development [82].

The duration of the clinical trial will be 25 months, with 12 months of recruitment period and 12 months of posttreatment follow up period.

**Fig 1:**
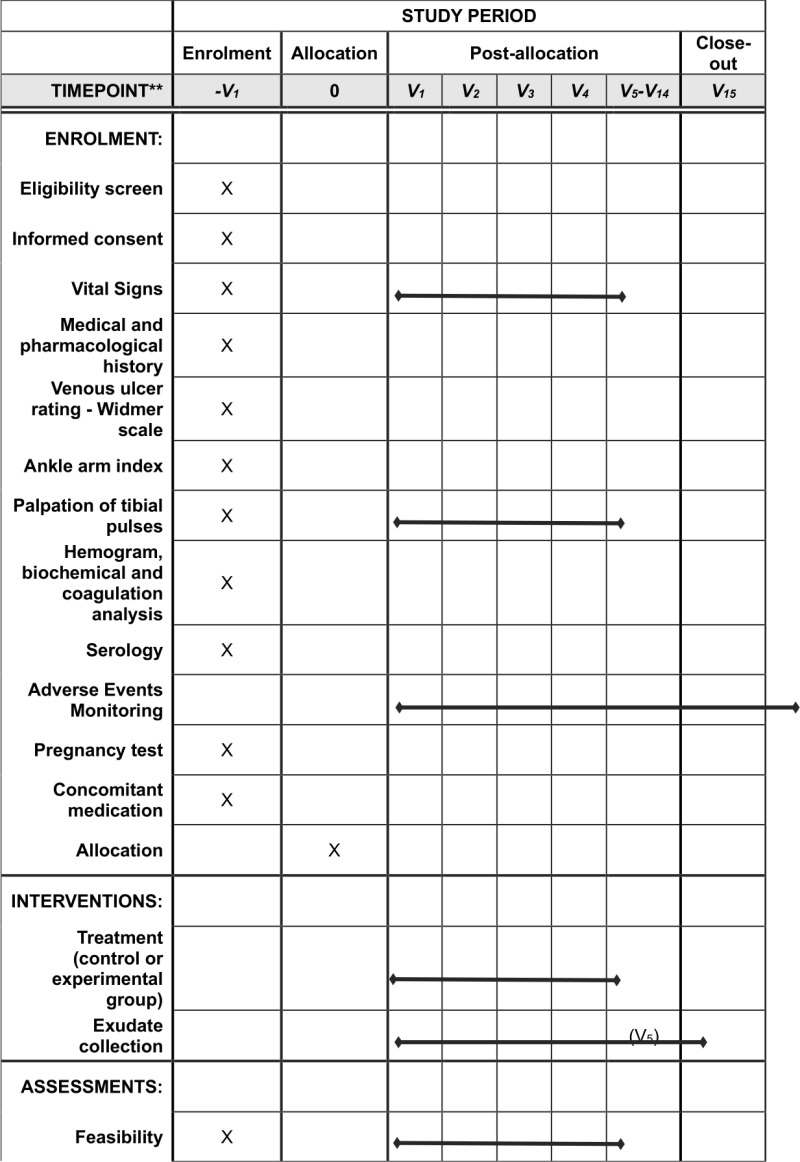
SPIRIT checklist. -V_1_. Enrolment visit. V_2_. Allocation visit. V_1_ – V_14_. Post – allocation visits. V_15_. Close out visit.

Patient recruitment will begin on April 14, 2024. The recruitment period is expected to conclude in April 2025, although a one-year extension (April 2026) will be requested if necessary. Data collection of the intervention phase and study of variables will be completed once the treatment of the last patient is finished (expected May 2026). Results should be available by September 2026. All details regarding enrolment, interventions, and assessments are outline in the Spirit schedule as shown in Fig 1.

### Inclusion criteria

In order to be included in the study, patients must meet all these inclusion criteria: a) signature of the informed consent (IC) after reading the patient information sheet (PIS); b) over 18 years of age of both genders; c) active or recurrent VLU in the lower extremity with an area between 5–10 cm^2^; d) grade III lesion classification measured by the Widmer scale (S3 File); e) palpable distal pulses in lower limbs [(tibial and pedal). Index ankle arm between 0.8–1.3].

### Exclusion criteria

Patients included in the study may not present any of the following exclusion criteria: a) any pathology for which the investigator considers compressive bandaging to be contraindicated and/or previous acute deep vein thrombosis (DVT) within 10 days of symptom onset; b) grade III obesity with a body mass index (BMI) >40 kg/m^2^; or underweight patients (BMI < 18.5 kg/m^2^); c) active neoplasia and/or in treatment with cytostatics; d) patients undergoing radiotherapy treatment in areas close to the lesion; e) clinical signs of colonization or local infection of the lesion; f) lymphangitis; g) chronic lymphoedema; h) venous ulceration grade I or II on the Widmer scale; i) lesions close to cancerous lesions; j) non – localized wounds on lower extremities; k) ongoing infection and/or sepsis; l) critical lower limb ischemia or other venous disease of unknown origin; m) immunosuppressed patients; n) dialysis patients; o) patients with thalassemia; p) decompensated heart failure; q) pregnant and lactating women; r) any other concurrent disease or condition that, in the opinion of the investigator, would render the patient unfit to participate in the study.

### Randomization

The randomization method is simple and unrestricted, using a table of random numbers. The randomization list is kept and managed by the promoter’s Coordination Unit (And&tAT), which assigns the treatment after confirming compliance with the selection criteria. To minimize potential bias in this study, several measures have been implemented, including strict allocation concealment. The allocation sequence was generated using a computer-based randomization process and securely managed by an independent researcher who was not involved in participant recruitment or data collection. This approach ensures that allocation assignments remain concealed from both investigators and participants until the point of intervention. Additionally, blinding procedures have been established to further reduce bias, including maintaining the anonymity of group assignments for outcome assessors and statisticians analyzing the data. These steps are designed to minimize selection and performance bias, thereby enhancing the validity and reliability of the study findings.

### Interventions

#### Experimental intervention.

In the experimental group, the intervention is carried out with the characteristics specified below (S1 File).

#### Investigational therapy.

- Investigational product: allogeneic adult mesenchymal stem cells from adipose tissue expanded in a biological matrix of fibrin – hyaluronic acid.
- Active substance: allogeneic adult expanded ATMSCs. MSCs from surgically excised donor adipose tissue meet all the necessary specifications, including the expression of characteristic surface markers and the ability to differentiate into cell lines of mesodermal origin. The MSCs have been produced under GMP conditions. Once in the laboratory, the tissue was washed with a solution consisting of Dulbecco’s phosphate buffered saline (DPBS), gentamicin and vancomycin. After this, in order to isolate the MSCs, mechanical disaggregation of the tissue was combined with enzymatic digestion. Once isolated, the cells were cultured for expansion at 37 ºC and 5% CO_2_. All human samples were obtained with the informed consent of the patient, as well as in accordance with the ethical standards of the committee responsible for human experimentation (Provincial Ethical Committees of Granada) and with the Helsinki Declaration of 1975, revised in 1983.

- Pharmaceutical form: live tissue equivalent.
- Route of administration: topical – dermal.

- Schedule: 4 doses. One matrix administered weekly. Each patient assigned to the experimental treatment group will receive 4 sheets at a concentration of about 360,000 cells/cm^2^. The BAMS has a surface area of 21 cm^2^ and contains a total of 7.5 x 10^6^ ATMSCs.
- Manufacturer of the investigational product: Cell Production and Tissue Engineering Unit (CPTEU) of the Hospital Universitario Virgen de las Nieves (Granada, España).

#### Procedure for administration and treatment in the experimental group.

First, exudate is collected from the wound for further study. After this, the following steps are performed:

- Cleaning and washing of the wound with saline solution NaCl 0.9% (NaCl 0.9%; BraumTM, 346056); exerting sufficient pressure to remove debris or detritus without damaging the wound tissue.
- If necessary, mechanical debridement of devitalized areas or areas without scar viability should be carried out in order to create a suitable environment to promote the healing process.
- To determine the area of the lesion, we will use a graduated ruler, measuring the width and length of the wound and will proceed with the calculation as indicated:

$$\text{Area}\;\text{=}\;\text{length}\;\text{x}\;\text{width}\;\text{=}\;\text{c}{\text{m}}^{\text{2}}$$

- Application of the BAMS on the wound bed. Placement shall be such that it covers the entire ulcer bed and the periphery. The prepared matrix shall be provided by CPTEU in sterile packaging. The professional in charge of placing the matrix will only have to adapt it to the area of the lesion.
- Wound coverage with a secondary non-adhesive polyurethane foam dressing (New Cutimed® Siltec Plus 73288/001) and Jobst^®^ Compri2^®^ 40 mmHg compression therapy.
- The day of the first treatment application will be considered day 0. From that moment, the same procedure will be performed every 7 days until completing 4 weeks of treatment. The weekly dose to be received is a 21 cm^2^ sheet with 7.5 x 10^6^ ATMSCs.

#### Control intervention.

Patients assigned to the control group will receive the same treatment, except for the application of the BAMS. In this case, the non-adhesive polyurethane foam dressing (New Cutimed^®^ Siltec Plus 73288/001) will not be considered secondary. They will also receive Jobst^®^ Compri2^®^ 40 mmHg compression therapy (S2 File).

#### Procedure for administration and treatment in the control group.

First, exudate is collected from the wound for further study. After this, the following steps are performed:

- Cleaning and washing of the wound with saline solution NaCl 0.9% (NaCl 0.9%; BraumTM, 346056); exerting sufficient pressure to remove debris or detritus without damaging the wound tissue.
- If necessary, mechanical debridement of devitalized areas or areas without scar viability should be carried out in order to create a suitable environment to promote the healing process.
- Determination of the wound area, as previously explained.
- Wound coverage with a non-adhesive polyurethane foam dressing (New Cutimed® Siltec Plus 73288/001) and Jobst^®^ Compri2^®^ 40 mmHg compression therapy.
- No advanced wound care measures, such as cellular or tissue-based therapies, bioengineered skin substitutes, or growth factor applications, will be used in the control group to ensure a clear comparison with the experimental intervention
- The day of the first treatment application will be considered day 0. From that moment, the same procedure will be performed every 7 days until completing 4 weeks of treatment. The weekly dose to be received is a 21 cm^2^ sheet with 7.5 x 10^6^ ATMSCs.

### Study time points

Throughout the clinical trial there will be 5 visits within the screening and treatment phases, followed by 9 safety and efficacy follow-up visits until day 84. Additionally, a variable number of safety follow-up visits will be scheduled up to 12 months after the last administration. The interventions planned at each visit will be conducted according to the trial outline in Table 1.

**Table 1:** Schedule of visits.

Visits	V 1	V 2	V 3	V 4	V 5	V 6	V 7	V 8	V 9	V 10	V 11	V 12	V 13	V14	V15 – until completion of follow – up 12 months after last infusion
**Identification of the visit**	Selection Randomization	1st Dose Day 0	2nd Dose Day + 7	3rd Dose Day + 14	4th Dose Day + 21	Follow-up visit Day + 28	Follow-up visit Day + 35	Follow-up visit Day + 42	Follow-up visit Day + 49	Follow-up visit Day + 56	Follow-up visit Day + 63	Follow-up visit Day + 70	Follow-up visit Day + 77	Follow-up visit Day + 84	**Post – treatment safety follow – up (every 15 days)**
**Informed Consent**	X														
**Demographic data and lifestyle habits**	X														
**Vital Signs**	X	X	X	X	X	X	X	X	X	X	X	X	X	X	
**Medical and pharmacological history**	X														
**VU rating - Widmer scale**	X														
**Ankle arm index**	X														
**Palpation of tibial pulses**	X	X	X	X	X	X	X	X	X	X	X	X	X	X	
**Hemogram, biochemistry and coagulation**	X					X				X				X	
**Serology**	X														
**Resvech Scale**	X	X	X	X	X	X	X	X	X	X	X	X	X	X	
**MolecuLight i:X Wound Extension**	X	X	X	X	X	X	X	X	X	X	X	X	X	X	
**VAS Scale**	X	X		X		X		X		X		X		X	
**CIVIQ 20 Questionnaire**	X	X			X		X		X		X			X	
**Treatment of VU - BAMS**		X	X	X	X										
**Pregnancy test**	X														
**Sample Collection - Exudate Study**		X	X	X	X	X									
**Adverse events/ Mortality**		X	X	X	X	X	X	X	X	X	X	X	X	X	X
**Concomitant medication**	X	X	X	X	X	X	X	X	X	X	X	X	X	X	X

-V_1_. Enrolment visit. V_2_. Allocation visit. V_1_ – V_14_. Post – allocation visits. V_15_. Close out visit.

- Visit 1: Screening and randomization. Patients will be provided with detailed information about the clinical trial and will sign the informed consent form. Patient data will be recorded, including vital signs, ulcer classification (Widmer Scale), pulse palpation, blood analysis, pregnancy test, macroscopic wound assessment, wound area measurement, visual analogue pain scale (VAS), Venous Disease Quality of Life Questionnaire – 20 (CIVIQ20).

Once the inclusion criteria have been checked, the patient will be included in the study and the data will be sent to the ANdtAT sponsor for randomization.

- Visit 2. Treatment Visit (week 1 - day 0). Vital signs, palpation of pulses, macroscopic assessment of the lesion, measurement of wound area, Visual Analogic Scale (VAS scale), collection of exudate sample for biomolecular study, CIVIQ20 scale and VLU treatment (experimental or control) will be assessed. Record any changes in the patient’s clinical status or adverse events in the medical record.
- Visit 3. Treatment Visit (week 2 - day + 7). Vital signs, palpation of pulses, macroscopic assessment of the lesion, measurement of wound area, VAS scale, CIVIQ20 scale, collection of exudate sample for biomolecular study, VLU treatment (experimental or control). Record changes in the patient’s clinical status or adverse events in the medical record.
- Visit 4. Treatment visit (week 3 - day + 14). Vital signs, palpation of pulses, macroscopic assessment of the lesion, measurement of wound area, collection of exudate sample for biomolecular study, VAS scale, CIVIQ20 scale, VLU treatment (experimental or control). Record changes in the patient’s clinical status or adverse events in the medical record.
- Visit 5. Treatment visit (week 4 - day + 21). Vital signs, palpation of pulses, macroscopic assessment of the lesion, measurement of wound area, collection of exudate sample for biomolecular study, VAS scale, CIVIQ20 scale, VLU treatment (experimental or control). Record changes in the patient’s clinical status or adverse events in the medical record.
- Follow-up visits (until 12 months after the last treatment). Subsequently, once the patient has completed the 4 – week treatment period, 9 follow-up visits (visits 6–14) on efficacy and safety is performed at 42, 49, 56, 63, 70, 77 and 84 days from the beginning of treatment. This additional follow-up is focused on assessing the evolution of the lesion after treatment and the long – term safety of the BAMS.

### Data collection

#### Patient selection.

Preselection of subjects is performed by each physician/nurse responsible for the patient based on clinical and analytical findings during routine patient follow-up. After ensuring that the patient has understood the information regarding the trial, the purpose and methodology, and has signed the informed consent; the necessary procedures for confirmation that the patient meets all inclusion criteria, and no exclusion criteria will be performed.

#### Identification of subjects and confidentiality of data.

Patients are identified with a sequential alphanumeric code, which is assigned to each patient in sequential order of inclusion when they give their informed consent.

The sponsor can only identify subjects by the code assigned to them and their gender. The investigator is responsible for maintaining a record of the patient´s names and the assigned identification code.

Study data will be transcribed onto data collection forms each with a unique identification code assigned to the patient, which will serve as the identifier for the patients and their samples. Only doctors and nurses participating in the study and personnel authorized by official bodies, if necessary, will have access to the patients’ medical records in accordance with Organic Law 3/2018, of 5 December, on Personal Data Protection and the guarantee of digital rights (Spain).

This confidential information is owned by the principal investigator, and may it not be disclosed to others without prior written consent from the investigator. Furthermore, this information may only be for the purposes of this study. Any data generated during the study will also be considered confidential and will be used solely by the investigators for the study´s objectives and the development of the study drug.

#### Patient replacement policy.

All included patients who have withdrawn prior to randomization or after randomization, provided they have not received any doses of the BAMS, will be replaced. However, patient who withdraws after having received the allocated treatment, because of side effects or ineffectiveness of the treatment, shall not be replaced. For patient replacement, a mirror list will be developed in the randomization system.

#### Data collection notebook.

The investigators, or an authorized representative of the study team, must complete a case report form (CRF) for each patient enrolled in the study, including those who do not complete the study. If a patient withdraws from the study, the reason for withdrawal must be indicated on the DCN. In cases where withdrawal is due to a treatment – limiting adverse event, every effort should be made to clearly document the outcome.

#### Study monitoring.

Monitoring is performed by the And&tAT Coordination Unit. The responsible monitor will maintain regular contact with the investigator and conduct site visits to inspect trial records (DCN and other relevant data) and source documents, ensuring compliance with patient confidentiality in accordance with local requirements. The monitor is responsible for reviewing the DCN at regular intervals during the trial to verify adherence with the protocol and ensure that all recorded data are complete, consistent, and accurate. Additionally, the monitor will have access to laboratory test reports and other patient records as needed to verify the information included in the DCN. The investigator agrees to collaborate with the monitor to ensure that any problems detected during these monitoring visits are resolved. 100% of the data collected are susceptible to be monitored.

### Measurement of outcomes

#### Primary endpoints.

- Feasibility. Defined as the possibility of completing the administration of all 4 doses of the investigational medicinal product in at least 80% of the patients randomized to the treatment group.
- Safety. Incidence of adverse event, serious adverse event, adverse reaction, as well as serious unexpected adverse reaction, during the entire intervention period; as well as 12 months after the end of treatment. Adverse effects of trials to date using mesenchymal stem cell-based therapies in wound treatment have been collected as S4 File.

#### Secondary endpoints.

Efficacy. The response is analyzed through clinical and biological parameters.

#### Clinical.

- Percentage of patients achieving complete healing at 12 weeks follow-up (complete epithelialization and maintained for 2 weeks), when comparing control versus treatment group. Complete epithelialization will be assessed as the full closure of the wound, characterized by the absence of visible open areas, exudate, or granulation tissue, with the presence of intact epidermis covering the previous wound site. This assessment will be performed by trained clinicians using standardized wound measurement tools and photographic documentation, as described in previous studies on wound healing assessment [83]. For this clinical trial, the treatment would be considered effective if the experimental group achieves a significantly higher percentage of complete healing than the control group. The study will analyze these results statistically to ensure that any observed differences are clinically relevant and not due to random variation.
- Elapsed time (days) to wound closure, measured from the start of treatment to the absence of loss of skin continuity (time at which the wound bed is completely re – epithelialized and with new tissue), when comparing control versus treatment group. Assessment of time to wound closure will be measured by the rate at which treatments are applied and subsequent follow-ups. It will be assessed by visual examination with photographic recording and application of the Resvech scale.
- Percentage of patients showing improvement in RESVECH 2.0 (lesion size, depth - affected tissues, edges, wound bed tissue type, exudate, and infection – inflammation – (S5 File); when comparing control versus treatment. The RESVECH 2.0 will be used as a standard tool to objectively assess the wound characteristics and evolution of the patients included in the study. This scale includes dimensions such as lesion size, depth and affected tissues, edges, type of tissue in the wound bed, exudate, and infection/inflammation, allowing a personalised assessment of the healing process. RESVECH 2.0 is based on a Likert scale, where the lower the score the better the wound condition and the higher the score the worse the wound condition (minimum score 0/maximum score 35). A significant improvement will be considered if the RESVECH 2.0 score decreases by at least 30–50% from the initial score.
- Percentage reduction in wound extension (relative area), when comparing control versus experimental group.
- Percentage of patients with a decrease in VAS scale score, when comparing control versus experimental group. VAS is a tool used to measure pain intensity. It consists of a 10 cm line with “No pain” (0 cm) at one end and “Worst pain imaginable” (10 cm) at the other. The patient marks a point on the line to indicate their pain level, which is then measured in centimeters to provide a score between 0 and 10. The VAS is commonly used in clinical settings due to its simplicity and effectiveness in assessing pain.
- Percentage of patients perceiving improvement in quality of life according to the CIVIQ20 score throughout treatment/follow-up, comparing both groups. The CIVIQ-20 survey is a tool consisting of 20 questions distributed in 4 dimensions (physical, psychological, social function and pain). The questions are graded from 1 to 5 points (Likert-type), the score ranges from 20 to 100, where 20 is the best and 100 the worst quality of life. In this case, a decrease in the initial score on the CIVIQ-20 test of 5 or more points is considered as a target for improvement in quality of life.

#### Biological.

Evolution of the pattern of cytokines and growth factors obtained from ulcer exudate samples at day 0, +7, +14, +21 and +28, when comparing both groups and their relationship with ulcer healing. Significant changes in cytokine levels will be determined based on both absolute and relative variations from baseline values. A statistically significant difference (p < 0.05) between treatment groups will be considered clinically relevant. Reference values for inflammatory and pro-healing cytokines (such as IL-6, TNF-α, VEGF, and TGF-β) will be based on published literature [84] and compared to control values within our study.

### Ethical considerations

The clinical trial is authorized by the Spanish Agency for Medicines and Health Products (SAMHP) and the Granada Biomedical Research Ethics Committee (BREC) with approval granted on March 20, 2023.

The trial is conducted in accordance with the protocol following the sponsor’s standard operating procedures and those established at the participating health center.

It complies with the ethical principles outlined in the Declaration of Helsinki, as revised at successive World Medical Association Assemblies (WMA, 2013); as well as the current Spanish legislation on Clinical Trials (RD 1090/2015). Additionally, the guidelines of the standards of good clinical practice (ICH-GCP & CPMP/ICH/135/95) are followed.

The investigational product meets the definition of “Advanced therapy investigational medicinal product” as stated in Regulation (CE) nº 1394/2007 of the European Parliament and of the Council.

### Data analysis

Statistical analysis will be carried out by an independent biostatistician under the supervision of the principal investigator of the clinical trial, using SPSS 30.0.0. All continuous variables will be described using standardized statistical measures. Categorical variables will be summarized in frequency tables. Descriptive analyses will use absolute frequencies or relative frequencies in the case of relative variables. Quantitative variable will be shown as m ± SD (mean and standard deviation) and range (minimum and maximum) or P50 [P25 – P75] (median, interquartile range). To compare the different variables in the 2 groups, since the chi-square and Fisher’s tests are non-parametric, we will use Fisher’s exact test to compare the proportion of complete healing between the experimental and control groups, given the sample size of 20 patients. This method is more appropriate for small samples and allows for the evaluation of statistically significant differences in contingency tables with low expected frequencies. Since complete epithelization is a time-to-event analysis, we will use the Kaplan-Meier method to describe the evolution in both groups and the log-rank test for comparison, recognizing the potential limitation in statistical power due to the sample size. If the number of events allows, we will explore a Cox regression model adjusted for relevant covariates. To compare healing time between groups, we will consider a non-parametric test such as the Mann-Whitney test if required by the data. Patient improvement will be defined as a significant reduction in the total RESVECH 2.0 score from baseline to the end of treatment, with proportions of improvement compared between groups using Fisher’s exact test. Additionally, data normality will be assessed using the Shapiro-Wilk or Kolmogorov-Smirnov test. If the data follow a normal distribution, a two-way ANOVA will be applied to evaluate the effect of treatment and time; if normality is not met, the Friedman test will be used, as it is more suitable for non-parametric comparisons in repeated measures. To analyze pain reduction at multiple time points, a two-way repeated measures ANOVA (group × time) will be performed if normality and homogeneity of variance assumptions are met; otherwise, more robust alternatives such as a linear mixed model (LMM) or the Friedman test will be considered. Finally, to compare the final scores of the CIVIQ-20 scale between the intervention groups, the Kruskal-Wallis test will be used, while within-group comparisons of initial and final scores will be performed using the Friedman test.

Values with p < 0.05 will be considered statistically significant.

## Discussion

Several studies have shown the effect of MSCs on tissue regeneration. In terms of wound healing, MSCs exert a therapeutic effect through a combination of direct cellular interactions and the secretion of bioactive molecules that modulate the immune response. Their immunomodulatory properties are primarily mediated through the release of anti-inflammatory cytokines such as interleukin-10 (IL-10) and TGF-β, which suppress pro-inflammatory responses and encourage tissue regeneration [39,85]. Additionally, MSCs interact directly with immune cells, such as T cells, natural killer (NK) cells, and macrophages, modulating their activity to create an anti-inflammatory environment [86]. For example, MSCs induce the differentiation of regulatory T cells (Tregs) and polarize macrophages towards an M2 phenotype, which is associated with wound healing and tissue remodeling [45,87]. Moreover, extracellular vesicles (EVs) secreted by MSCs play a critical role in these processes by delivering microRNAs, proteins, and lipids to target cells, enhancing paracrine signaling and the repair of damaged tissues [40,88]. Additionally, recent studies have highlighted the ability of MSC-derived exosomes to mediate oxidative stress reduction and promote angiogenesis through VEGF signaling pathways [89]. Due to its properties, different products based on this cell therapy have been designed for the treatment of chronic wounds, that are difficult to manage and heal poorly.

The incidence and prevalence of chronic VLU wounds pose a major economic and healthcare challenge, impacting patients’ well-being, increasing the workload of healthcare professionals, and driving up healthcare costs. In addition, the therapeutic approach to chronic wounds varies widely, often lacking uniformity, leading to frequent changes in treatment strategies due to ineffectiveness.

This clinical trial explores the generation of a biologically based therapy aimed at addressing the underlying factors contributing to the chronicity of these wounds. Thus, BAMS therapy has the potential to accelerate VLU healing, improve patients’ quality of life and pain levels, reduce the burden on healthcare professionals, and lower overall healthcare costs. One of the key challenges in the clinical application of MSCs for wound healing is the variability in patient response. Factors such as age, underlying comorbidities, and genetic differences can influence how effectively MSCs promote tissue regeneration, leading to inconsistent outcomes across patients. Furthermore, the therapeutic efficacy of MSCs may be affected by difficulties in maintaining the stability of the BAMS preparations. The process of isolating and expanding MSCs, as well as the preservation and handling conditions, can impact cell viability and functionality, which in turn influences the success of treatment. This variability in MSC quality, along with challenges in standardizing manufacturing protocols, highlights the need for optimized techniques to enhance the consistency and reproducibility of MSC-based therapies. Addressing these issues will be crucial for ensuring that BAMS can achieve predictable and sustained wound healing outcomes across diverse patient populations.

On the other hand, in our study protocol, we have established clear guidelines for handling amendments to the trial, including potential early termination. Any modifications to the study design, procedures, or outcomes will be subject to approval by the institutional review board (IRB). These amendments will be carefully documented, and all investigators will be promptly informed. If safety concerns arise or interim analysis suggests futility or significant adverse effects, participants will be informed, and appropriate measures will be taken to ensure their safety and well-being. Additionally, any significant changes to the protocol will be communicated in updated versions of the trial registry (NCT05962931) and the final publication to ensure full transparency.

## Limitations

The limitations presented by this clinical trial are mainly based on the number of patients included in each intervention arm. Despite this, it should be noted that this is a proof-of-concept clinical trial. Thus, to enhance the robustness and generalizability of the study, consideration of a multi-center design is warranted. A multi-center approach would enable the inclusion of a more diverse patient population, accounting for variations in demographics, clinical practices, and healthcare environments that could influence treatment outcomes. Such a design would also strengthen the external validity of the findings by demonstrating the intervention’s efficacy across different settings and healthcare systems. Although this study is currently designed as a single-center trial due to logistical and resource constraints, future phases of research will prioritize the incorporation of a multi-center framework to ensure broader applicability and to address potential variability in treatment effects. This progression will be pivotal in advancing the clinical translation and adoption of the intervention.

On the other hand, we acknowledge the potential limitations associated with simple randomization using a random number table, as highlighted by the reviewer. While this method ensures randomness in the allocation process, we recognize that it may not fully account for imbalances in baseline characteristics among treatment groups, which could introduce bias. To address this concern, stratified or block randomization could be implemented in future studies to enhance the reliability of the results by ensuring a more balanced distribution of key confounding variables. This approach would allow for a more rigorous evaluation of the intervention’s effects, particularly in larger trials or multi-center designs.

Another limitation is the stability of BAMS and the secondary dressing for one week. In this regard, different studies with preparations like the one in this clinical trial have shown that the biological dressings used are stable for more than one week.

## Conclusions

Currently, there is ample evidence on wound healing therapies that focus on producing early closure of VLUs and avoiding the complications of injury as efficiently as possible. However, there are no studies that confirm the usefulness of this biological dressing (BAMS) in the treatment of chronic wounds through their effects on the healing process.

BAMS based therapy could be effective in the management of VLUs, reducing wound closure time and the occurrence and associated complications of these skin lesions. Another positive impact, could be the improvement of the quality of life of patients suffering from these wounds, based on the regenerative and analgesic properties of this biological dressing, its ability to differentiate to different cell lines, the promotion of the secretion of certain growth factors involved in cell proliferation and angiogenesis (VEGF, TGFβ1 and HGF), as well as the accumulation of extracellular matrix at the site of healing.

## Supporting information

S1 File Standard investigational drug administration procedure (BAMS). (PDF)

S2 File Standard procedure for the treatment of uncomplicated venous ulcers. (PDF)

S3 File Widmer scale. (PDF)

S4 File Adverse effects of the use of mesenchymal stem cells in wounds. (PDF)

S5 File RESVECH 2.0 scale. (PDF)

S6 File Original protocol. (PDF)
