# Supplementary material for: Application of allogeneic adult mesenchymal stem cells in the treatment of venous ulcers: A phase I/II randomized controlled trial protocol
Source: PLoS One. 2025 May 15;20(5):e0323173. doi: 10.1371/journal.pone.0323173 (PMC12080757; doi:10.1371/journal.pone.0323173)
Supplement: S1 File — (PDF) [file pone.0323173.s001.pdf]

Supporting information 1. Standard investigational drug administration procedure (BAMS).

1. Remove the compressive therapy and, subsequently, the dressing covering the lesion. Perform the technique carefully taking care not to damage the wound bed or perilesional skin.
2. Exudate sample collection with filter paper (FisherbrandTM, Fisher Scientific S.L., Madrid, Spain):
    a. Exudate samples will be taken from the ulcer bed by absorption with filter paper (500 – 1000 μl).
    b. Place the filter paper over the lesion. It is only necessary to place it, it is not necessary to rub.
    c. One minute after placing the absorbent paper, it shall be placed in 0.5mL of cold phosphate buffered saline solution.
    d. The samples will be stored at a minimum temperature of -20ºC until analysis.
3. Cleaning and lavage of the wound with normal saline (NaCl 0.9%), exerting sufficient pressure to flush out debris or detritus without damaging the wound tissue.
4. Mechanical debridement of devitalized areas or areas without scar viability:
    a. Carefully scrub the area of the lesion on which you wish to perform the technique.
    b. Drag away non – viable tissue debris
    c. Perform a second cleansing of the lesion with normal saline (NaCl 0.9%), exerting sufficient pressure so that debris or detritus is washed away without damaging the wound tissue.
5. Determine the wound area using a graduated ruler.
6. Deposit the BAMS on the base of the wound depending on the size of the lesion.
    a. The material necessary for the handling and administration of the matrix is as follows:
        - Sterile drape
        - Sterile gloves
        - Sterile gauze
        - Saline solution (NaCl 0.9%), if necessary.
        - Sterile blunt-tipped scissors
        - Simple sterile dissecting fórceps
        - Sterile demographic marker (Ref. 400133181).
    b. The BAMS must be kept cold at all times (2 - 8ºC).
    c. Remove the mesh from the refrigerator.
    d. Ensure a sterile field before handling the matrix

e. Keep in mind the area of the lesion to calculate the necessary portion of matrix.
f. Open the container where the mesh is deposited once sterility is guaranteed.
g. With the help of the demographic marker and the ruler included in the same package, mark the area of matrix you need according to the area of the lesion.
h. Trim, if necessary, the matrix to fit the wound area.
i. Carefully pick up the portion of matrix with the help of the simple dissecting forceps.
j. Deposit the piece of matrix on the wound without exerting pressure, ensuring that it covers the lesion correctly and that it remains in contact with the wound bed.

7. Cover the wound with a secondary non-adhesive polyurethane foam dressing (New Cutimed® Siltec Plus 73288/001).
8. Jobst® Compri2® 40mmHg multilayer compressive therapy of the site of injury.
