# Supplementary material for: Application of allogeneic adult mesenchymal stem cells in the treatment of venous ulcers: A phase I/II randomized controlled trial protocol
Source: PLoS One. 2025 May 15;20(5):e0323173. doi: 10.1371/journal.pone.0323173 (PMC12080757; doi:10.1371/journal.pone.0323173)
Supplement: S2 File — (PDF) [file pone.0323173.s002.pdf]

Supporting Information 2. Standard procedure for the treatment of uncomplicated venous ulcers.

1. Remove the compressive therapy and, subsequently, the dressing covering the lesion. Perform the technique carefully taking care not to damage the wound bed or perilesional skin.
2. Exudate sample collection: with filter paper (FisherbrandTM, Fisher Scientific S.L., Madrid, Spain):
    a. Exudate samples will be collected from the ulcer bed by absorption with filter paper (500 – 1000 μl).
    b. Place the filter paper over the lesion. It is only necessary to place it, it is not necessary to rub.
    c. Within one minute of placing the absorbent paper, it should be placed in 0.5mL of cold phosphate buffered saline solution.
    d. The samples will be stored at a minimum temperature of -20ºC until analysis.
3. Cleaning and lavage of the wound with normal saline (NaCl 0.9%), exerting sufficient pressure to flush out debris or detritus without damaging the wound tissue.
4. Mechanical debridement of devitalized areas or areas without scar viability:
    a. Gently scrub the area of the lesion on which you wish to perform the technique.
    b. Drag away non-viable tissue debris
    c. Perform a second cleansing of the lesion with normal saline (NaCl 0.9%), exerting sufficient pressure so that debris or detritus is washed away without damaging the wound tissue.
5. Determine the wound area with the aid of a graduated ruler.
6. Cover the wound with a hydrofiber dressing (Aquacel Extra®; 480228).
7. Traditional Compressive Therapy (Jobst® Compri2®; BDF0232)
