# Supplementary material for: Application of allogeneic adult mesenchymal stem cells in the treatment of venous ulcers: A phase I/II randomized controlled trial protocol
Source: PLoS One. 2025 May 15;20(5):e0323173. doi: 10.1371/journal.pone.0323173 (PMC12080757; doi:10.1371/journal.pone.0323173)
Supplement: S3 File — (PDF) [file pone.0323173.s003.pdf]

Supporting information 3. Widmer scale

Wound stage	Clinical signs
I	Edema, subfascial congestion, phlebectasia, varicose veins
II	Induration, pigmentation, eczema
III	Ulcer, ulcerous scar
