# Supplementary material for: Application of allogeneic adult mesenchymal stem cells in the treatment of venous ulcers: A phase I/II randomized controlled trial protocol
Source: PLoS One. 2025 May 15;20(5):e0323173. doi: 10.1371/journal.pone.0323173 (PMC12080757; doi:10.1371/journal.pone.0323173)
Supplement: S4 File — (PDF) [file pone.0323173.s004.pdf]

Supporting Information 4. Adverse effects of completed clinical trials of mesenchymal stem cells for ulcer treatment.

**Type of study/ Author and year**	**Type of injury**	**Cell type**	**Administration guideline**	**Adverse Events**
Konstantinow et al. 2017	Venous ulcers and mixed venous and arterial ulcers	Autologous adipose tissue mesenchymal stromal Cells (ADSCs)	1 suspension of ADSCs injected intradermally at a depth of 5-10 mm into the centre and edges of the wound.	No serious adverse events (SAEs) were reported. Short-term moderate bruising and donor site discomfort were reported in 5 patients receiving oral anticoagulants.
Aycan et al. 2018	Venous ulcers	Autologous ADSCs	20 mL ADSCs injected intradermally into and around the wound	No Adverse Events (AEs) such as infection, inflammation or tissue reaction were observed.
RCT / Hashemi et al. 2019	Diabetic ulcers	Allogeneic Wharton´s Jelly mesenchymal stem cells (WJ-MSCs)	3 doses of an acellular amniotic membrane containing WJ-MSCs. Topical use.	N/D
RCT phase II/ Zollino et al. 2018	Venous ulcers	Unexpanded Autologous ADSCs	1 dose of ADSCs (high concentration of cells in a 1mL syringe) injected intradermally 1 cm deep into the ulcer bed and edges.	No SAEs were reported. One case of perilesional dermatitis was reported shortly after injection of the cells, which resolved spontaneously in the second week.
RCT/ Moon et al. 2019	Diabetic ulcers	Allogenic ADSCs	5x5cm hydrogel film for topical use	There were no SAEs related to cell treatment. There were no significant differences between the 2 treatment groups. The incidence of SAEs was similar between the treatment and control groups.
Maslowski et al. 2020	Venous ulcers	Autologous ADSCs	A suspension of ADSCs in a volume of 4.5 mL administered in multiple injections under and around the ulcer.	There were no SAEs. One patient had minor swelling of no clinical significance.
RCT/ Tanios et al., 2021	Chronic diabetic ulcers, venous, trophic and ischaemic ulcers	Autologous ADSCs	ADSCs suspension, every 3 weeks, intravenously	N/D

RT phase I/Zhang et al. 2022	Diabetic ulcers	Allogeneic umbilical cord mesenchymal stem cells (UC-MSCs)	1 topical (subcutaneous) dose 2 intravenous doses at a dose of $2x10^5$ cells/Kg with an upper limit of $1x10^7$cells	2 cases of transient fever were considered to be possibly related to intravenous transfusion of UC-MSCs
RT phase I/IIa /Kerstan et al. 2022	Diabetic ulcers	Allogeneic skin-derived ABCB5+ MSCs	2 doses of 2×10^6 ABCB5+ MSC (suspended in a solution of lactated Ringer's solution containing 2.5% albumin and 0.4% glucose) / cm^2. Topical use.	83 AEs were reported. 9 AEs reported by 7 patients were SAEs, 2 of which (heart failure and pulmonary embolism) resulted in death. Only 3 AEs: increased wound exudation (mild), erythema (moderate) and venous ulcer pain (moderate), were related to treatment. These events resolved without sequelae.
RCT phase I/II /Arango et al. 2022	Diabetic ulcers	Allogeneic Bone Marrow Mesenchymal Stem Cells (BM MSC) and allogeneic BM MSC derivatives	2 doses of BM MSC derivatives (1mL) or 1 dose of BM MSC ($1x10^6$ cells) injected intradermally around wounds	No AEs were reported
RT phase I/ Asko et al. 2022	Diabetic ulcers	BM allogeneic MSCs enriched in CD362	1 dose of allogeneic BM MSC suspension enriched in CD362 in a collagen solution (10.6×10^6 CMTMo/3 cm2). 1mL. Topical use	7 AEs and 1 SAE occurred. Both patients experienced increased drug-related exudation from the treated ulcer. However, it resolved without complications within one week of drug application. The SAEs consisted of neurological symptoms, which resulted in hospital admission. However, the SAEs were not related to the drug.
RCT/ Falanga et al. 2022	Venous ulcers	Allogenic BM MSC	BM MSC skin spray in fibrin (1 million cels/cm2) Topical use.	No SAEs were reported

REFERENCES:

Konstantinow, A. et al. Therapy of ulcus cruris of venous and mixed venous arterial origin with autologous, adult, native progenitor cells from subcutaneous adipose tissue: a prospective clinical pilot study. J. Eur. Acad. Dermatology Venereol. 31, (2017).

Kavala, A. & Turkyilmaz, S. Autogenously derived regenerative cell therapy for venous leg ulcers. Arch. Med. Sci. – Atheroscler. Dis. 3, 156–163 (2018).

Hashemi, S. S. et al. The healing effect of Wharton's jelly stem cells seeded on biological scaffold in chronic skin ulcers: A randomized clinical trial. J. Cosmet. Dermatol. 18, (2019).

Zollino, I. et al. A phase II randomized clinical trial for the treatment of recalcitrant chronic leg ulcers using centrifuged adipose tissue containing progenitor cells. Cytotherapy 21, 200–211 (2019).

Moon, K. C. et al. Potential of allogeneic adipose-derived stem cell–hydrogel complex for treating diabetic foot ulcers. Diabetes 68, 837–846 (2019).

Masłowski, L. et al. Autotransplantation of the Adipose Tissue-Derived Mesenchymal Stromal Cells in Therapy of Venous Stasis Ulcers. Arch. Immunol. Ther. Exp. (Warsz). 68, 5 (2020).

Tanios, E. et al. Efficacy of adipose-derived stromal vascular fraction cells in the management of chronic ulcers: a randomized clinical trial. Regen. Med. 16, 975–988 (2021).

Zhang, C. et al. Topical and intravenous administration of human umbilical cord mesenchymal stem cells in patients with diabetic foot ulcer and peripheral arterial disease: a phase I pilot study with a 3-year follow-up. Stem Cell Res. Ther. 13, 1–14 (2022).

Tappenbeck, N. et al. In vivo safety profile and biodistribution of GMP-manufactured

Becerra-Bayona, S. M. et al. Mesenchymal stem cells derivatives as a novel and potential therapeutic approach to treat diabetic foot ulcers. Endocrinol. Diabetes Metab. Case Reports 2020, (2020).

Askø Andersen, J. et al. Novel topical allogeneic bone-marrow-derived mesenchymal stem cell treatment of hard-to-heal diabetic foot ulcers: a proof of concept study. Stem Cell Res. Ther. 13, 1–9 (2022).

Falanga, V. et al. Autologous Cultured Bone Marrow-Derived Mesenchymal Stem Cells in a Fibrin Spray to Treat Venous Ulcers: A Randomized Controlled Double-Blind Pilot Study. Surg. Technol. Int. 40, 47–54 (2022).
