# Supplementary material for: Application of allogeneic adult mesenchymal stem cells in the treatment of venous ulcers: A phase I/II randomized controlled trial protocol
Source: PLoS One. 2025 May 15;20(5):e0323173. doi: 10.1371/journal.pone.0323173 (PMC12080757; doi:10.1371/journal.pone.0323173)
Supplement: S5 File — (PDF) [file pone.0323173.s005.pdf]

Supporting information 5. Resvech scale

Items	Measurement 0	Measurement 1	Measurement 2	Measurement 3
**1. Size of the lesion**				
0. Surface=0cm^2				
1. Surface<4 cm^2				
2. Surface4≤16cm^2				
3. Surface=16≤36 cm^2				
4. Surface=36≤64cm^2				
5. Surface=64≤100cm^2				
6. Surface≥100 cm^2				
**2. Depth/Tissues affected**				
0. Intact healed skin				
1. Dermis-epidermis involvement				
2. Subcutaneous tissue involvement (adipose tissue without reaching the muscle fascia)				
3. Muscle involvement				
4. Involvement of bone and/or adjoining tissues (tendons, ligaments, joint capsule or black eschar that does not allow the tissues underneath to be seen).				
**3. Wound edges**				
0. Not distinguishable				
1. Diffuse				
2. Delimited				
3. Damaged				
4. Engrossed				
**4. Type of tissue in the wound bed**				
4. Necrotic (dry or wet black eschar)				
3. Necrotic tissue and/or sloughing on the bed				
2. Granulation tissue				
1. Epithelial tissue				
0. Closed/Cicatrization				
**5. Exudate**				
3. Dry				
0. Wet				
1. Very wet				
2. Saturated				
3. With exudate leakage				
**6. Infection/Inflammation (Biofilm signs)**				
6.1. Increasing pain (Yes=1/No=0)				

6.2. Erythema at perilesion (Yes=1/No=0)				
6.3. Edema in the perilesion (Yes=1/No=0)				
6.4. Increase in temperature (Yes=1/No=0)				
6.5. Increasing exudate (Yes=1/No=0)				
6.6. Purulent exudate (Yes=1/No=0)				
6.7. Tissue friable or bleeding easily (Yes=1/No=0)				
6.8. Stagnant wound, not progressing (Yes=1/No=0)				
6.9. Biofilm-compatible tissue (Yes=1/No=0)				
6.10. Smell (Yes=1/No=0)				
6.11. Hypergranulation (Yes=1/No=0)				
6.12. Increased wound size (Yes=1/No=0)				
6.13. Satellite lesions (Yes=1/No=0)				
6.14. Tissue pallor (Yes=1/No=0)				

TOTAL SCORE: maximum 35 points / minimum 0 (the higher the score, the worse the condition of the lesion)
