# Supplementary material for: Application of allogeneic adult mesenchymal stem cells in the treatment of venous ulcers: A phase I/II randomized controlled trial protocol
Source: PLoS One. 2025 May 15;20(5):e0323173. doi: 10.1371/journal.pone.0323173 (PMC12080757; doi:10.1371/journal.pone.0323173)
Supplement: S6 File — (PDF) [file pone.0323173.s006.pdf]

Phase I/II Proof-of-Concept, Multicenter, Randomized, Controlled Clinical Trial to assess the feasibility, safety, and efficacy of mesenchymal cell application using adult allogenic stem cells derived from adipose tissue, expanded in a biological matrix of fibrin-hyaluronic acid, for the treatment of venous ulcers in the lower limbs.

Protocol code: UV/AP/21

Nº EudraCT: 2021-001341-12

Amendment nº 2

Version of July 15, 2024

Promoter: Andalusian Network of Design and Translation in Advanced Therapies -

Fundaciôn Pública Andaluza Progreso y Salud M.P.

This protocol will be carried out in accordance with the ICH-GCP (CPMP/ICH/135/95), the ethical principles of the Declaration of Helsinki and the Spanish legislation in force (RD 1090/2015).

INDEX

1 SUMMARY 11

1.1. TITLE 11

1.2. TRIAL IDENTIFICATION 11

1.3. COORDINATING RESEARCHER OF THE STUDY 11

1.4. PROMOTER 11

1.5. DRUG RESEARCH ETHICS COMMITTEE THAT EVALUATED THE STUDY 11

1.6. STUDY MONITOR 11

1.7. RESEARCH PRODUCT 12

1.8. CLINICAL TRIAL PHASE 12

1.9. MAIN OBJECTIVE OF THE STUDY 12

1.10. STUDY DESIGN 12

1.11. MAIN VARIABLE 12

1.12. STUDY POPULATION AND TOTAL NUMBER OF PATIENTS 12

1.13. DURATION OF THE STUDY 13

1.14. SCHEDULE AND EXPECTED COMPLETION DATE 13

1.15. UNIT IN CHARGE OF THE PRODUCTION OF THE PEI 13

1.16. INDEPENDENT COMMITTEE ON DATA SECURITY AND MONITORING 13

1.17. RECRUITMENT EVALUATION COMMITTEE 14

1.18. PRINCIPAL INVESTIGATORS AND SITES PLANNED TO CONDUCT THE STUDY 14

2. JUSTIFICATION 15

2.1. INTRODUCTION 15

2.2. REFERENCES 16

3. HYPOTHESIS 22

4. CLINICAL TRIAL OBJECTIVES 22

4.1. MAIN OBJECTIVE 22

4.2. SECONDARY OBJECTIVES 22

5. TYPE OF CLINICAL TRIAL AND DESIGN 23
5.1. DEVELOPMENT PHASE 23
5.2. DESIGN 23
5.3. SAMPLE SIZE 23
5.4. DESCRIPTION OF THE TREATMENT. 23
5.5. CRITERIA FOR NON-RESPONSE 24
6. SUBJECT SELECTION 24
6.1. STUDY POPULATION AND TOTAL NUMBER OF PATIENTS 24
6.2. IDENTIFICATION OF THE SUBJECTS AND CONFIDENTIALITY OF THE DATA 24
6.3. INCLUSION CRITERIA 25
6.4. EXCLUSION CRITERIA 25
7. EVALUATION VISITS 26
8. TREATMENT ASSIGNMENT 33
8.1. RANDOMIZATION 33
8.2. WITHDRAWAL CRITERIA AND PLANNED ANALYSIS OF WITHDRAWALS AND ABANDONMENTS 33
8.3. PATIENT REPLACEMENT POLICY 34
8.4. TRIAL INTERRUPTION 34
9. DESCRIPTION OF THE TREATMENT 34
9.1. INVESTIGATIONAL THERAPY 34
9.2. COMPARATOR 35
9.3. PERMITTED AND PROHIBITED CONCOMITANT TREATMENTS 35
9.3.1. PERMITTED CONCOMITANT TREATMENTS 35
9.3.2. PROHIBITED CONCOMITANT TREATMENTS 36
9.4. INVESTIGATIONAL PRODUCT IDENTIFICATION 36
9.5. PROCEDURE FOR ADMINISTERING THE PEI 36
9.6. HEALING PROCEDURE IN THE CONTROL GROUP 36
10. EVALUATION OF THE RESPONSE 37

10.1. MAIN VARIABLE 37
10.2. SECONDARY VARIABLES 37
11. STATISTICAL ANALYSIS 38
12. SAFETY RATING 38
12.1. ADVERSE EVENTS 38
12.2. DEFINITIONS 39
12.3. ASSESSMENT OF THE CAUSALITY AND SEVERITY OF AAS 41
12.4. PREGNANCIES 42
12.5. RECORDING AND REPORTING OF ADVERSE EVENTS 42
12.5.1. NON-SERIOUS ADVERSE EVENTS 42
12.5.2. SERIOUS ADVERSE EVENTS 43
12.5.3. OVERDOSE 44
12.6. EXPEDITED NOTIFICATION OF RAGI 44
13. ASPECTS ETHICAL 44
13.1. CONSIDERATIONS GENERAL 44
13.2. STUDY MONITORING 45
13.3. RETENTION OF STUDY DOCUMENTATION 45
13.4. TRIAL INSURANCE 46
14. DATA COLLECTION NOTEBOOKS 46
15. FOLLOW-UP AND FINAL REPORTS AND COMMUNICATION OF RESULTS 46
16. CONDITIONS FOR PUBLICATION OF RESULTS 46
ANNEX 1: SCHEDULE OF VISITS 48
ANNEX 2: STANDARD PROCEDURE FOR ADMINISTRATION OF THE INVESTIGATIONAL PRODUCT. 50
ANNEX 3: STUDY OF EXUDATE 52
ANNEX 4: STANDARD PROCEDURE FOR THE CURE OF UNCOMPLICATED VENOUS ULCERS. 53
ANNEX 5: BASIC CEAP CLASSIFICATION 54
ANNEX 6: RESVECH 2.0 SCALE 55

ANNEX 7: WIDMER CLASSIFICATION 57

ANNEX 8: HELSINKI DECLARATION 58

ANNEX 9. PATIENT IDENTIFICATION CARD MODEL 65

1 SUMMARY

1.1. TITLE

Phase I/II, proof-of-concept, multicenter, randomized, controlled clinical trial to assess the feasibility, safety, and efficacy of the application of adult mesenchymal stem cells derived from adipose tissue, expanded in a biological matrix of fibrin-hyaluronic acid, for the treatment of venous ulcers in the lower limbs.

1.2. TRIAL IDENTIFICATION

Nº EudraCT: 2021-001341-12

Protocol code: UV/AP/21

Version of 15 July 2024

1.3. COORDINATING RESEARCHER OF THE STUDY

Dr. Víctor Javier Costela Ruiz.

Granada Health District Metropolitan, Andalusian Health Service

University of Granada, Department of Nursing, Faculty of Health Sciences

1.4. PROMOTER

Andalusian Network for the Design and Translation of Advanced Therapies- Andalusian Public Foundation for Progress and Health.

1.5. DRUG RESEARCH ETHICS COMMITTEE THAT EVALUATED THE STUDY

Research Ethics Committee of Granada, Network of Research Ethics Committees of the Andalusian Public Health System.

1.6. STUDY MONITOR

The monitoring will be carried out by personnel belonging to the Coordination Unit of the Promoter itself.

1.7. RESEARCH PRODUCT

Name: Expanded allogeneic adult mesenchymal stem cells from adipose tissue (ADSCs) in a biological matrix of fibrin- hyaluronic acid.

Product name: BAMS*, Bioengineered Artificial Mesenchimal Sheet.*

Pharmaceutical form: Living tissue equivalent.

Administration: Topic.

1.8. CLINICAL TRIAL PHASE

Phase I/II.

1.9. MAIN OBJECTIVE OF THE STUDY

1. To evaluate the feasibility of cutaneous application of ADSCs in a biological matrix of fibrin-hyaluronic acid on venous ulcers (UV).
2. To examine the safety of cutaneous administration of ADSCs in a biological matrix of fibrin-hyaluronic acid over UVs compared to conventional treatment.

1.10. STUDY DESIGN

Phase I/II, multicenter, randomized, controlled, open-label clinical trial.

1.11. MAIN VARIABLE

Feasibility

- Defined as the possibility of completing the administration of the 4 doses of the Investigational Drug in at least 80% of patients randomized to the treatment group.

Safety:

- Incidence of AE, SAE, AR, as well as SUAR, throughout the intervention period in patients in the experimental group, as well as in the 12 months after the end of treatment, compared to the control group.

1.12. STUDY POPULATION AND TOTAL NUMBER OF PATIENTS

20 patients with venous ulcers (VU) from the Primary Care Units of the Granada and Metropolitan District will be included.

1.13. DURATION OF THE STUDY

The duration of the clinical trial will be 43 months.

Recruitment period: 30 months.

Follow-up period after treatment: 12 months.

1.14. SCHEDULE AND EXPECTED COMPLETION DATE

Estimated start date: 12 September 2024.

Clinical trial end date: The end of the trial is defined as the date on which the last trial visit of the last patient recruited takes place.

1.15. UNIT IN CHARGE OF THE PRODUCTION OF THE PEI

Cell Production and Tissue Engineering Unit of the Virgen de las Nieves Hospital in Granada (UPCIT)

1.16. INDEPENDENT COMMITTEE ON DATA SECURITY AND MONITORING

An *ad hoc* Independent Data Security and Monitoring Committee (DSMC) will be constituted for this trial, consisting of 4 members.

The profile of the 4 members will be as follows:

- ✓ Two clinical consultants: one of them an expert in the therapeutic area in which the clinical trial is carried out,
- ✓ A clinical methodologist,
- ✓ An expert in ethical matters.

The functions of DSMC will include:

- ✓ To guarantee the correct inclusion of patients, acting as a consulting body when the sponsor raises doubts in this regard.
- ✓ Advise in doubtful cases on the withdrawal of patients from the clinical trial.

- ✓ Supervision of periodic safety reviews.
- ✓ Advise on any issues affecting patient safety.
- ✓ Participate in any decision that serves to ensure that the trial is carried out in accordance with Good Clinical Practice, including recommendations to the sponsor on the advisability of continuing, modifying or stopping the trial.

The responsibilities of DSMC members shall be documented in a standard work plan which shall be prepared and approved in advance by the sponsor prior to its submission to each of the Committee Members. Likewise, the frequency of the meetings and how the monitoring of the security data will be carried out will be recorded.

The list of members of the DSMC is contained in the document: Composition of the Security and Data Monitoring Committee.

1.17. RECRUITMENT EVALUATION COMMITTEE

An Independent Internal Committee for the evaluation of *ad hoc* recruitment will be set up to ensure that selection criterion 3 is met: "Active or recurrent venous ulcer (C6 according to the CEAC clinical classification, Annex 5) in the lower extremity with an area of between 5-10 cm^2"

The trial sponsor, after receiving information from the recruiting center, will be responsible for obtaining agreement from the Committee members regarding compliance with this criterion, according to a Standard Operating Procedure drafted *ad hoc*.

1.18. PRINCIPAL INVESTIGATORS AND SITES WHERE THE STUDY IS PLANNED

Ms. Encarnaciôn González Vigil

Ms. Mª Ángeles Granado Contrelas

Ms. María Teresa Martínez Lao

Ms. Ana María Baena

It is an authentic©copy of an electronic

2. JUSTIFICATION

2.1. INTRODUCTION

According to data from the National Consensus Conference on Lower Extremity Ulcers (NCCLEU), in Spain 75-80% of lower extremity ulcers are venous in etiology, with a prevalence of 0.5 to 0.8% and an incidence of 2 to 5 per thousand people/year (1).

There are circumstances that complicate the approach to this type of wound: the long period of monitoring, rapid evolution or the frequent occurrence of events that complicate its resolution (colonization by microorganisms, comorbidity, age, etc.) (2-4).

Scientific evidence shows the usefulness of adult trunk mesenchymal cells of adipose tissue (ADSCs) in the healing of different chronic wounds such as diabetic foot, ulcers and burns, among others. ADSCs have analgesic properties and accelerate healing by the secretion of factors with action in angiogenesis [vascular endothelial growth factor (VEGF)], the migration of endogenous progenitor cells to the focus, such as endothelial cells; the production of extracellular matrix, the ability to differentiate into certain cell groups such as fibroblasts, myofibroblasts and keratinocytes; as well as certain immunosuppressive effects that they confer anti-inflammatory properties (5-31). On this last property, the ADSCs, through a mechanism known as polarization of mesenchymal stem cells, are able to suppress immune response in cytokine-rich inflammatory microenvironments, such as in the case of chronic wounds. Thus, in the event of elevated levels of cytokines and/or proinflammatory factors such as IL-6, interferon gamma (IFN-γ) or tumour necrosis factor-alpha (TNF-α). Mesenchymal stem cells also produce anti-inflammatory and regenerative factors such as transforming growth factor beta (TGF-β) or hepatocyte growth factor (HGF) (32-34).

An important aspect to keep in mind is the economics of health and injuries. In this way, several studies have shown that there are four important variables to be considered in the assessment: prognosis, economic cost, quality of life, and cost savings (32). The annual cost of treating wounds such as pressure ulcers at the national level is estimated to range from 461 to 602 million € (33). In England, annual costs in the treatment of UVs are estimated to be about £1938 (34). Between 1.5 and 3% of the total budget of the National Health Systems of the European Union is dedicated to direct and indirect costs in the treatment of ulcers of any etiology. In the case of venous ulcers, the cost when the healing period is less than 12 weeks is 400 to 500 €; in the case of ulcers healed between 12 weeks and 6 months, 900 to 1000 €. It is important to bear in mind that a venous ulcer that does not heal within a period of 4 weeks increases the costs of treatment in the case of a venous disease that does not heal.

It is an authentic©copy of an electronic

comparison with those that heal within 4 weeks (35-38). Thus, bearing in mind the hypothesis of acceleration in the healing process and regeneration with the treatment that is the subject of this trial; although it is an expensive treatment, the expense derived from the treatment of this type of injury will be considerably reduced.

The high incidence of UVs, the time required in the therapeutic approach to them, the high frequency in which standard therapy is not effective, the high health cost, the loss of the quality of life of the sufferer, together with scientific evidence on the effectiveness of regenerative therapies by means of ADSCs (1,3,34,39-44), justifies the use of this therapy to improve healing capacity, affecting the patient's quality of life and avoiding overload in the health system at different levels.

The objective of this project is to evaluate the therapeutic efficacy/effectiveness and safety of the local administration of expanded adult adult stem mesenchymal cells of adipose tissue (ADSCs) in the biological matrix of fibrin-hyaluronic acid in the treatment of UVs.

Therefore, we proposed a phase I/II clinical trial (CE), multicentric and randomized, in a sample of patients affected by this type of lesions in the provincial area of Granada. The study population will be randomly distributed according to a computer program distribution into two groups: experimental and control. From visit 2 to 5, the experimental group will receive locally one sheet of ADSCs in a biological fibrin-hyaluronic acid matrix per week, for a total of four. The control group will receive the conventional treatment. Patients in both groups will be subjected to the same wound care and follow-up protocol. Data will be collected on feasibility and safety, as well as response (tissue factors related to the healing process, exudate, depth, affected surface, etc.), healing or closure time, as well as aspects related to safety.

2.2. REFERENCES

1. Casanova PL. National Consensus Conference on Lower Extremity Ulcers [Internet]. GNEAUPP. 2014 [cited 2020 August 27]. Available at: https://gneaupp.info/conferencia- national-consensus-on-lower-extremity-ulcers/

2. Collins L, Seraj S. Diagnosis and treatment of venous ulcers. Am Fam Physician. April 15, 2010; 81(8):989-96.

3. Spear M. Venous ulcers--an evidence-based update. Plast Surg Nurs. December 2012; 32(4):185- 8.

It is an authentic©copy of an electronic

4. Ramsay S, Cowan L, Davidson JM, Nanney L, Schultz G. Wound samples: moving towards a standardised method of collection and analysis. International Wound Journal. 2016; 13(5):880-91.

5. Vall§e M, Côt§ JF, Fradette J. Adipose-tissue engineering: Taking advantage of the properties of human adipose-derived stem/stromal cells. Pathologie Biologie. June 1, 2009; 57(4):309-17.

6. Ebrahimian TG, Pouzoulet F, Squiban C, Buard V, Andr§ M, Cousin B, et al. Cell therapy based on adipose tissue-derived stromal cells promotes physiological and pathological wound healing. Arterioscler Thromb Vasc Biol. April 2009; 29(4):503-10.

7. Nambu M, Kishimoto S, Nakamura S, Mizuno H, Yanagibayashi S, Yamamoto N, et al. Accelerated wound healing in healing-impaired db/db mice by autologous adipose tissue-derived stromal cells combined with atelocollagen matrix. Ann Plast Surg., March 2009; 62(3):317-21.

8. Han SK, Kim HR, Kim WK. The treatment of diabetic foot ulcers with uncultured, processed lipoaspirate cells: a pilot study. Wound Repair Regen. August 2010; 18(4):342-8.

9. Yun IS, Jeon YR, Lee WJ, Lee JW, Rah DK, Tark KC, et al. Effect of human adipose derived stem cells on scar formation and remodeling in a pig model: a pilot study. Dermatol Surg., October 2012; 38(10):1678-88.

10. Hassan WU, Greiser U, Wang W. Role of adipose-derived stem cells in wound healing. Wound Repair Regen. June 2014; 22(3):313-25.

11. Amato B, Compagna R, Amato M, Butrico L, Fugetto F, Chibireva MD, et al. The role of adult tissue- derived stem cells in chronic leg ulcers: a systematic review focused on tissue regeneration medicine. International Wound Journal. 2016; 13(6):1289-98.

12. Raposio E, Bertozzi N, Bonomini S, Bernuzzi G, Formentini A, Grignaffini E, et al. Adipose-derived Stem Cells Added to Platelet-rich Plasma for Chronic Skin Ulcer Therapy. Wounds. April 2016; 28(4):126-31.

13. Jiang X, Zhang H, Teng M. Effectiveness of Autologous Stem Cell Therapy for the Treatment of Lower Extremity Ulcers: A Systematic Review and Meta-Analysis. Medicine (Baltimore). March 2016; 95(11):e2716.

14. Lee DE, Ayoub N, Agrawal DK. Mesenchymal stem cells and cutaneous wound healing: novel methods to increase cell delivery and therapeutic efficacy. Stem Cell Res Ther. 2016 Mar 9;7:37 AM

It is an authentic©copy of an electronic

15. Athanerey A, Patra PK, Kumar A. Mesenchymal stem cell in venous leg ulcer: An intoxicating therapy. J Tissue Viability. August 2017; 26(3):216-23.

16. Konstantinow A, Arnold A, Djabali K, Kempf W, Gutermuth J, Fischer T, et al. Therapy of ulcus cruris of venous and mixed venous arterial origin with autologous, adult, native progenitor cells from subcutaneous adipose tissue: a prospective clinical pilot study. J Eur Acad Dermatol Venereol. December 2017; 31(12):2104-18.

17. Motegi SI, Ishikawa O. Mesenchymal stem cells: The roles and functions in cutaneous wound healing and tumor growth. J Dermatol Sci. May 2017; 86(2):83-9.

18. Motegi SI, Sekiguchi A, Uchiyama A, Uehara A, Fujiwara C, Yamazaki S, et al. Protective effect of mesenchymal stem cells on the pressure ulcer formation by the regulation of oxidative and endoplasmic reticulum stress. Sci Rep. 07 of 2017; 7(1):17186.

19. Chopinaud M, Labb§ D, Creveuil C, Marc M, B§nateau H, Mourgeon B, et al. Autologous Adipose Tissue Graft to Treat Hypertensive Leg Ulcer: A Pilot Study. Dermatology (Basel). 2017; 233(2-3):234-41.

20. Carstens MH, Gômez A, Cort§s R, Turner E, P§rez C, Ocon M, et al. Non-reconstructable peripheral vascular disease of the lower extremity in ten patients treated with adipose-derived stromal vascular fraction cells. Stem Cell Res. 2017;18:14-21.

21. Klar AS, Zimoch J, Biedermann T. Skin Tissue Engineering: Application of Adipose-Derived Stem Cells. Biomed Res Int. 2017;2017:9747010.

22. Uchiyama A, Motegi SI, Sekiguchi A, Fujiwara C, Perera B, Ogino S, et al. Mesenchymal stem cells-derived MFG-E8 accelerates diabetic cutaneous wound healing. J Dermatol Sci. June 2017; 86(3):187-97.

23. Gadelkarim M, Abushouk AI, Ghanem E, Hamaad AM, Saad AM, Abdel-Daim MM. Adipose-derived stem cells: Effectiveness and advances in delivery in diabetic wound healing. Biomed Pharmacother. 2018 Nov;107:625-33.

24. Guo J, Hu H, Gorecka J, Bai H, He H, Assi R, et al. Adipose-derived mesenchymal stem cells accelerate diabetic wound healing in a similar fashion as bone marrow-derived cells. Am J Physiol Cell Physiol. December 1, 2018; 315(6):C885-96.

It is an authentic©copy of an electronic

25. Holm JS, Toyserkani NM, Sorensen JA. Adipose-derived stem cells for treatment of chronic ulcers: current status. Stem Cell Res Ther [Internet]. 2018 May 15 [cited 2020 August 25];9. Available in: https:// www.ncbi.nlm.nih.gov/pmc/articles/PMC5952370/

26. Yoon D, Yoon D, Sim H, Hwang I, Lee JS, Chun W. Accelerated Wound Healing by Fibroblasts Differentiated from Human Embryonic Stem Cell-Derived Mesenchymal Stem Cells in a Pressure Ulcer Animal Model. Stem Cells Int [Internet]. 2018 Dec 30 [cited 2020 Aug 23];2018. Available in: https:// www.ncbi.nlm.nih.gov/pmc/articles/PMC6332923/

27. Huang TY, Wang GS, Tseng CC, Su WT. Epidermal cells differentiated from stem cells from human exfoliated deciduous teeth and seeded onto polyvinyl alcohol/silk fibroin nanofiber dressings accelerate wound repair. Mater Sci Eng C Mater Biol Appl. 2019 Nov;104:109986.

28. Torres-Torrillas M, Rubio M, Damia E, Cuervo B, Del Romero A, Peláez P, et al. Adipose-Derived Mesenchymal Stem Cells: A Promising Tool in the Treatment of Musculoskeletal Diseases. Int J Mol Sci. June 25, 2019; 20(12).

29. Moon KC, Suh HS, Kim KB, Han SK, Young KW, Lee JW, et al. Potential of Allogeneic Adipose-Derived Stem Cell-Hydrogel Complex for Treating Diabetic Foot Ulcers. Diabetes. 2019; 68(4):837-46 .

30. Hashemi SS, Mohammadi AA, Kabiri H, Hashempoor MR, Mahmoodi M, Amini M, et al. The healing effect of Wharton's jelly stem cells seeded on biological scaffold in chronic skin ulcers: A randomized clinical trial. Journal of Cosmetic Dermatology. 2019; 18(6):1961-7.

31. Becerra-Bayona SM, Solarte-David VA, Sossa CL, Mateus LC, Villamil M, Pereira J, et al. Mesenchymal stem cells derivatives as a novel and potential therapeutic approach to treat diabetic foot ulcers. Endocrinol Diabetes Metab Case Rep. 2020 July 5;2020.

32. Ge W, Jiang J, Baroja ML, Arp J, Zassoko R, Liu W, et al. Infusion of mesenchymal stem cells and rapamycin synergize to attenuate alloimmune responses and promote cardiac allograft tolerance. Am J Transplant. August 2009; 9(8):1760-72.

33. Heo JS, Kim S. Human adipose mesenchymal stem cells modulate inflammation and angiogenesis through exosomes. Sci Rep. February 17, 2022; 12(1):2776.

34. Bernardo ME, Fibbe WE. Mesenchymal stromal cells: sensors and switchers of inflammation. Cell Stem Cell. October 3, 2013; 13(4):392-402.

It is an authentic©copy of an electronic

35. Soldevilla Agreda JJ, Torra i Bou JE, Posnett J, Verdú Soriano J, San Miguel L, Mayan Santos JM. An approximation of the impact of the economic cost of the treatment of pressure ulcers in Spain. Gerokomos. December 2007; 18(4):43-52.

36. Al-Gharibi KA, Sharstha S, Al-Faras MA. Cost-Effectiveness of Wound Care: A concept analysis. Sultan Qaboos Univ Med J. November 2018; 18(4):E433-9.

37. Phillips CJ, Humphreys I, Thayer D, Elmessary M, Collins H, Roberts C, et al. Cost of managing patients with venous leg ulcers. International Wound Journal. 2020; 17(4):1074-82.

38. Chan B, Cadarette S, Wodchis W, Wong J, Mittmann N, Krahn M. Cost-of-illness studies in chronic ulcers: a systematic review. J Wound Care. April 1, 2017; 26(sup4):S4-14.

39. Guest JF, Ayoub N, McIlwraith T, Uchegbu I, Gerrish A, Weidlich D, et al. Health economic burden that different wound types impose on the UK s National Health Service. Int Wound J. April 2017; 14(2):322-30.

40. Lal BK. Venous ulcers of the lower extremity: Definition, epidemiology, and economic and social burdens. Semin Vasc Surg. March 2015; 28(1):3-5.

41. Rice JB, Desai U, Cummings AKG, Birnbaum HG, Skornicki M, Parsons N. Burden of venous leg ulcers in the United States. J Med Econ. May 2014; 17(5):347-56.

42. Rubio-Terr§s C, Dom«nguez-Gil Hurl§ A. Cost-effectiveness analysis of patient treatment with venous ulcers due to chronic venous insufficiency with purified and micronized flavonoic fraction and compressive therapy or with compressive therapy alone. Rev Esp Econ Salud. 2005; 4(2):87-94.

43. National Consensus Conference on Lower Extremity Ulcers: CONUEI consensus document. Barcelona: EdikaMed; 2009.

44. Green J, Jester R, McKinley R, Pooler A. The impact of chronic venous leg ulcers: a systematic review. J Wound Care. December 2, 2014; 23(12):601-12.

45. Roldán Valenzuela A, P§rez Barreno D, Ibáñez Clemente P, Navarro Caballero MA, Alba Moratilla C, Esparza Imas G, et al. Clinical Practice Guide. Consensus on Vascular Ulcers and Diabetic Foot of the Spanish Association of Vascular and Wound Nurses (AEEVH) [Internet]. SPANISH ASSOCIATION OF VASCULAR AND WOUND NURSING; 2017. Available in:
https:// www.seapaonline.org/UserFiles/File/Ulceras/Guia-de-Practica-Clinica-web.pdf

It is an authentic©copy of an electronic

46. Joaquim FL, Silva RMCRA, Garcia-Caro MP, Cruz-Quintana F, Pereira ER. Impact of venous ulcers on patients quality of life: an integrative review. Brazilian Journal of Nursing. August 2018; 71(4):2021-9.

47. Millan SB. Venous Ulcers: Diagnosis and Treatment. VENOUS ULCERS. 2019; 100(5):8.

48. Lurie F, Passman M, Meisner M, Dalsing M, Masuda E, Welch H, et al. The 2020 update of the CEAP classification system and reporting standards. J Vasc Surg Venous Lymphat Disord. May 2020; 8(3):342-52.

49. Serrano IR. The challenge of the Good Manufacturing Practice Standards specific to advanced therapy medicinal products in the European Union. 2019;6.

50. Zajdel A, Kałucka M, Kokoszka-Mikołaj E, Wilczok A. Osteogenic differentiation of human mesenchymal stem cells from adipose tissue and Whartons jelly of the umbilical cord. Acta Biochim Pol. 2017; 64(2):365-9.

51. Munir H, Ward LSC, Sheriff L, Kemble S, Nayar S, Barone F, et al. Adipogenic Differentiation of Mesenchymal Stem Cells Alters Their Immunomodulatory Properties in a Tissue:Specific Manner. Stem Cells. June 2017; 35(6):1636-46.

52. Sierra-Sánchez Á, Fernández-González A, Lizana-Moreno A, Espinosa-Ibáñez O, Martinez-Lopez A, Guerrero-Calvo J, et al. Hyaluronic acid biomaterial for human tissue-engineered skin substitutes: Preclinical comparative in vivo study of wound healing. J Eur Acad Dermatol Venereol. March 15, 2020;

53. Blasco Vera MÁ, Garc«a LA, Ort« PB, Romero IR, Sanfelix AH. Wound measurement systems. Rev enferm vasc. July 15, 2019; 2(4):17-21.

It is an authentic©copy of an electronic

3. HYPOTHESIS

Currently, the evidence on healing therapies is extensive, aimed at achieving early closure of venous ulcers (VU) and efficiently preventing complications arising from the injury. However, there are few studies confirming the effectiveness of ADSCs in the treatment of chronic wounds due to their impact on the healing process.

The hypothesis of this clinical trial is that therapy based on ADSCs could be effective in managing VUs by reducing wound closure time and the occurrence of associated complications, thereby improving the quality of life for affected patients. This is based on their regenerative and analgesic properties, as well as their ability to differentiate into various cell lines, promote the secretion of specific growth factors involved in cell proliferation and angiogenesis (such as Vascular Endothelial Growth Factor [VEGF] and Transforming Growth Factor β1 [TGFβ1]), along with Hepatocyte Growth Factor (HGF), and facilitate the accumulation of extracellular matrix at the healing site.

4. CLINICAL TRIAL OBJECTIVES

4.1. MAIN OBJECTIVE

- To evaluate the feasibility of the cutaneous application of ADSCs in the biological matrix of fibrin-hyaluronic acid on UVs.
- To examine the safety of cutaneous administration of ADSCs in a biological matrix of fibrin-hyaluronic acid over UVs compared to conventional treatment.

4.2. SECONDARY OBJECTIVES

1. To determine the effect of experimental treatment on wound closure time compared to the control group.
2. To assess the effect of the experimental treatment on the scar evolution of the injury, considering depth, dimensions, type of edges, tissue and exudate compared to the control group.
3. To assess the effect of the experimental treatment on the concentration of growth factors closely related to wound healing (cytokines IL-6, IL-4, TGF-β1 and IL-10) on the exudate of injury at day 0, +7, +14, +21 and +28 compared to the control group and its possible relationship with the rest of the efficacy parameters.

It is an authentic©copy of an electronic

4. To evaluate the evolution of pain derived from the presence of UVs in the group treated with the ADSCs, compared to the control group.

5. To determine the perceived quality of life after treatment with the experimental treatment compared to the control group.

5. TYPE OF CLINICAL TRIAL AND DESIGN

5.1. DEVELOPMENT PHASE

Phase I/II

5.2. DESIGN

Multicenter, randomized, controlled, open-label proof-of-concept clinical trial.

5.3. SAMPLE SIZE

Twenty patients with VUs, who meet all inclusion criteria and none exclusion criteria, will be included and will be randomized 1:1 to two intervention arms: experimental and control (10 patients treatment group/10 patients control group).

The 10 patients in the experimental arm will receive 4 doses of the investigational drug: adult mesenchymal stem cells from adipose tissue expanded in the biological matrix of fibrin-hyaluronic acid on the base or wound bed, while the 10 patients in the control group will be treated with conventional therapy.
This is a phase I/II clinical trial, proof of concept, of which there are no previous studies, so no mathematical calculation of the sample size has been carried out.

5.4. DESCRIPTION OF THE TREATMENT.

Phase I/II clinical trial, proof-of-concept, multicenter, randomized, controlled and open in a representative sample of patients affected by this type of lesions in Granada.

The study population will be randomly distributed according to a distribution computer program in two groups: experimental and control (1:1). The experimental group will receive four doses of ADSCs locally in the matrix format of fibrin · hyaluronic acid on a weekly basis, while the control group will receive conventional therapy. Patients in both study groups will be subjected to the same wound care and follow-up protocol.

It is an authentic©copy of an electronic

5.5. CRITERIA FOR NON-RESPONSE

Any AE/SAE that in the judgment of the investigator prevents the patient from receiving the next dose and/or forces the patient to withdraw the slide and change treatment, will be considered a failure/non-response criterion.

6. PATIENT SELECTION

The pre-selection of patients will be carried out by each physician/nurse responsible for the patient in view of the clinical and analytical results during the usual follow-up of the patients. After ensuring that the patient has understood the information regarding the trial, the purpose and methodology of the trial, and has signed the informed consent, the necessary procedures will be carried out to confirmation that the patient meets all inclusion criteria and none exclusion criteria. Once pre-selected, the patient will be referred to one of the nursing professionals who are members of the research team to continue with the selection process and subsequent intervention if necessary.

It is important to note that the profile of the patient who presents this pathology is usually a patient of elderly and with reduced mobility, who in some cases can live in nursing homes. Given the characteristics of this type of patient, it will be contemplated whether the Principal Investigator deems it necessary to treat the Investigational Medicinal Product at home or in a residential centre, maintaining the necessary measures for the correct transport of the product from the Health Centre to the home and/or residential centre; as well as for the maintenance of sterility.

6.1. STUDY POPULATION AND TOTAL NUMBER OF PATIENTS

20 patients from the Primary Care Units (UAP) of the Granada District with VU who meet all the inclusion criteria and none of the exclusion criteria will be included.

6.2. IDENTIFICATION OF THE SUBJECTS AND CONFIDENTIALITY OF THE DATA

Patients will be identified with a sequential alphanumeric code, which will be assigned to each patient according to the sequential order of inclusion when they give their informed consent. In the case that a patient is included in the study, that is, signs the informed consent, and once the procedures of the selection visit have been carried out, is considered unfit to continue in the study.

It is an authentic©copy of an electronic

The sponsor may only identify subjects by the code assigned to them, and their sex. The investigator should keep a record with the names of the patients and the identification code assigned.

The study data will be transcribed into data collection forms that will only include a code assigned to each patient, which will serve as an identification code for patients and samples.
Only doctors and nurses participating in the study and personnel authorized by official bodies, if necessary, may have access to the patients' medical records according to
Organic Law 3/2018, of 5 December, on the Protection of Personal Data and guarantee of digital rights.

This confidential information shall be the sole property of the principal investigator, may not be disclosed to others without the prior written consent of the investigator, and may not be used except for the prior written consent of the principal investigator.
for the realization of this study. The information that is created during the conduct of this clinical study is also considered confidential and will be used by the investigators in relation to the objectives of the study and the development of the study drug.

6.3. INCLUSION CRITERIA

To be eligible for inclusion in the study, patients must meet all the inclusion criteria described below:

1. Signing the informed consent (IC) after reading the patient information sheet (HIP).
2. Over 18 years of age of both sexes.
3. Active or recurrent venous ulcer (C6 according to the CEAC clinical classification, Annex 5) in the lower extremity with an area of between 5-10 cm^2.
4. Grade III on the Widmer scale.
5. Distal pulses in palpable lower limbs (tibial and pedium). ABI between 0.8 - 1.3.

6.4. EXCLUSION CRITERIA

Patients enrolled in the study may not meet any of the following exclusion criteria:

1. Any pathology for which the investigator considers that compression bandage is contraindicated and/or previous acute deep vein thrombosis (DVT), within the first 10 days from the onset of symptoms. The following comorbidities will be allowed:

It is an authentic©copy of an electronic

peripheral vascular disease, coronary heart disease, chronic kidney disease, chronic liver disease and arterial hypertension.

2. Grade III obesity with a body mass index (BMI) >40; or patients with insufficient weight (BMI <18.5).
3. Neoplasm active and/or under treatment with cytostatics.
4. Patients undergoing radiotherapy treatment in areas close to the injury.
5. Clinical signs of colonization or local infection of the injury.
6. Lymphangitis in the limb to be treated.
7. Chronic lymphedema in the limb to be treated.
8. Grade I or II venous ulcer on the Widmer scale.
9. Lesions close to possible or diagnosed cancerous lesions.
10. Non-localized wounds in the lower extremities.
11. Ongoing systemic infection.
12. Critical ischemia in the lower limbs or other venous diseases of unknown origin.
13. Immunosuppressed patients.
14. Dialysis patients.
15. Patients with thalassemia.
16. Decompensated heart failure.
17. Pregnant and breastfeeding women.
18. Any other concurrent disease or condition that, in the opinion of the investigator, would render the patient ineligible to participate in the study.

7. EVALUATION VISITS

Throughout the clinical trial, 5 visits will be carried out within the selection and treatment phases, 9 safety and efficacy follow-up visits up to day 84, and a variable number of safety follow-up visits until the completion of 12 months of follow-up after the last administration. The examinations planned for each of the visits will be carried out in accordance with the test scheme included in Annex 1.

It is an authentic©copy of an electronic

In case of need, due to deterioration or loss of the cure or the material used in it, the patient will go to the reference center to perform the procedure again. Care should be taken to maintain the secondary dressing, which will be in contact with the preparation and with the wound bed; it will be replaced, in case of deterioration or loss.

Visit 1. Selection and randomization visit

The selection of candidate patients will be carried out in the Primary Care Units selected as centers in which the study is planned to be carried out.

During this visit, patients must provide informed consent prior to performing any of the study procedures. It must be recorded in the clinical record that the patient agrees to participate in the clinical trial with his/her corresponding code, date of signature, as well as a justification for compliance with each of the selection criteria. At this time, the investigator will inform the sponsor of his or her inclusion in the study.

The date of the screening visit must be on or after the date of obtaining informed consent. This visit involves a clinical assessment of the candidates and information on the characteristics of the essay.

The following shall be recorded in the patient's medical record:

1. Date of the visit.
2. Demographic data (date of birth, sex) and clinical data (medical history, concomitant medication and lifestyle habits).
3. Vital signs (blood pressure, heart rate).
4. VU classification using the Widmer scale.
5. Correct palpation of tibial and pedial pulses. Blood test (biochemistry, blood count and serology).
6. Blood count (hemoglobin, leukocytes, lymphocytes, neutrophils and platelets), biochemistry (creatinine, bilirubin, GOT, GPT, GGT, albumin, triglycerides and total cholesterol) and coagulation (PT, PTT and fibrinogen).
7. Serology (HBV, HCV, HIV and syphilis).
8. Pregnancy test (if applicable).
9. Evaluation of the ulcer:

   A) Macroscopic observation of the injury: epithelialization tissue, presence of exudate and existence of bleeding using the Resvech scale.

It is an authentic©copy of an electronic

B) Wound extension by means of a graduated ruler, measuring the greatest vertical length (length) and the longest perpendicular length (width). Photographs will be taken with the ruler located on the injury to evaluate the evolution of the wound and the measurement of the perimeter.

10. Visual analogue pain scale (VAS).
11. Assessment of quality of life using the Venous Disease Quality of Life Questionnaire-20 (CIVIQ 20).
12. Mini Nutritional Assessment Test (MNA) Scale

Once it has been verified that all the inclusion criteria and none of the exclusion criteria are met, the patient can continue in the study. Otherwise, it will be considered a selection error, and the reason must be documented in the clinical history.

The patient's data will be sent to the sponsor who will be in charge of randomizing the patients. In
At the time this has been carried out, the patient will be contacted for the start of treatment at the Primary Care Center and the schedule of visits for the entire patient will be provided.
I am a student. The availability of the fibrin matrix with hyaluronic acid with ADSCs will be 48-72 hours from the communication by the promoter of the patient's randomization to the UPCIT. As described in previous sections, the treatment of patients will be 4 weeks. Patients assigned to the control group will receive the conventional therapy described by protocol and will have the same schedule of visits as the experimental group.

Visit 2. Treatment Visit (Week 1 - Day 0)

1. Date of the visit.
2. Vital signs (blood pressure, heart rate).
3. Correct palpation of tibial and pedial pulses.
4. Evaluation of the ulcer:

   A) Macroscopic observation of the injury: epithelialization tissue, presence of exudate and existence of bleeding using the Resvech scale.

   B) Wound extension by graduated ruler, measuring the longest vertically
   (length) and the longest perpendicular length (width). Photographs will be taken with the ruler located on the injury to evaluate the evolution of the wound and the measurement of the perimeter.

5. Visual Analogic Pain Scale (VAS).
6. Sample collection for the exudate study (Annex 3).
7. Evaluation of quality of life using the CIVIQ 20 questionnaire.

It is an authentic©copy of an electronic

8. VU treatment (Annex 2/Annex 4).

9. It will be recorded in the medical record if there has been any concomitant change in your medication since the last visit.

10. It will be recorded in the clinical history if any adverse event or death has occurred since the last visit.

Visit 3. Treatment Visit (Week 2 - Day +7)

1. Date of the visit.

2. Vital signs (blood pressure, heart rate).

3. Correct palpation of tibial and pedial pulses.

4. Evaluation of the ulcer:

   A) Macroscopic observation of the injury: epithelialization tissue, presence of exudate and existence of bleeding using the Resvech scale.

   B) Wound extension by graduated ruler, measuring the longest vertically (length) and the longest perpendicular length (width). Photographs will be taken with the ruler located on the injury to evaluate the evolution of the wound and the measurement of the perimeter.

5. VU treatment (Annex 2/Annex 4).

6. Sample collection for the exudate study (Annex 3).

7. It will be recorded in the medical record if there has been any concomitant change in your medication since the last visit.

8. It will be recorded in the clinical history if any adverse event or death has occurred since the last visit.

Visit 4. Treatment Visit (Week 3 – Day +14)

1. Date of the visit.

2. Vital signs (blood pressure, heart rate).

3. Correct palpation of tibial and pedial pulses.

4. Evaluation of the ulcer:

   A) Macroscopic observation of the injury: epithelialization tissue, presence of exudate and existence of bleeding using the Resvech scale.

It is an authentic©copy of an electronic

B) Wound extension by means of a graduated ruler, measuring the greatest vertical length (length) and the longest perpendicular length (width). Photographs will be taken with the ruler located on the injury to evaluate the evolution of the wound and the measurement of the perimeter.

5. Sample collection for the exudate study (Annex 3).
6. Visual Analogic Pain Scale (VAS).
7. VU treatment (Annex 2/Annex 4).
8. It will be recorded in the medical record if there has been any concomitant change in your medication since the last visit.
9. It will be recorded in the clinical history if any adverse event or death has occurred since the last visit.

Visit 5. Treatment Visit (Week 4 – Day +21)

1. Date of the visit.
2. Vital signs (blood pressure, heart rate).
3. Correct palpation of tibial and pedial pulses.
4. Evaluation of the ulcer:
   A) Macroscopic observation of the injury: epithelialization tissue, presence of exudate and existence of bleeding, using the Resvech scale.
   B) Wound extension by means of a graduated ruler, measuring the greatest vertical length (length) and the longest perpendicular length (width). Photographs will be taken with the ruler located on the injury to evaluate the evolution of the wound and the measurement of the perimeter.
5. Sample collection for the exudate study (annex 3).
6. Evaluation of quality of life using the CIVIQ 20 questionnaire.
7. VU treatment (Annex 2/Annex 4).
8. It will be recorded in the medical record if there has been any concomitant change in your medication since the last visit.
9. It will be recorded in the clinical history if any adverse event or death has occurred since the last visit.

It is an authentic©copy of an electronic

Follow-up visits (up to 12 months after the last treatment)

Subsequently, once the patient has completed the 4-week treatment period, 9 efficacy and safety follow-up visits (visits 6 - 14) will be made at 42, 49, 56, 63, 70, 77 and 84 days from the start of treatment.

In the event that patients living in nursing homes have been included in the trial, the follow-up visits, once the 4-week period of treatment has ended, may be carried out if the principal investigator considers it necessary, in the residence itself, as has been done during the usual follow-up of these patients.

This additional follow-up will focus on evaluating the progression of the injury after treatment and the long-term safety of ADSCs.

The follow-up visit to the evolution of the injury will be structured as follows:

1. Date of the visit.
2. Vital signs (blood pressure, heart rate).
3. Blood test (hemoglobin, leukocytes, lymphocytes, neutrophils, and platelets), biochemistry (creatinine, bilirubin, GOT, GPT, GGT, albumin, triglycerides, and total cholesterol) and coagulation (PT, PTT, and fibrinogen) (Visit 14).
4. Correct palpation of tibial and pedia pulses.
5. Evaluation of the ulcer:
   A) Macroscopic observation of the injury: epithelialization tissue, presence of exudate and existence of bleeding, using the Resvech scale.
   B) Wound extension by means of a graduated ruler, measuring the greatest vertical length (length) and the longest perpendicular length (width). Photographs will be taken with the ruler located on the injury to evaluate the evolution of the wound and the measurement of the perimeter.
6. Visual Analogic Pain Scale (VAS) (At visits 6, 7, 8, 9, 10, 11, 12, 13 and 14).
7. Sample collection for the exudate study (Annex 3) (Visit 6).
8. Assessment of quality of life using the CIVIQ 20 questionnaire (At visits 6, 7, 8, 9, 10, 11, 12, 13 and 14).
9. VU treatment (Annex 4).
10. It will be recorded in the medical record if there has been any concomitant change in your medication since the last visit.

It is an authentic©copy of an electronic

11. It will be recorded in the clinical history if any adverse event or death has occurred since the last visit.

From visit 15 onwards, two types of visits may be carried out depending on the state of the ulcer under study:

- If the ulcer is not healed, a weekly safety and efficacy follow-up will be carried out until it is completely closed. The following information will be collected on these visits:

1. Date of visit
2. Evaluation of the ulcer:
   A. Macroscopic observation of the injury: epithelialization tissue, presence of exudate and existence of bleeding, using the Resvech scale.
   B. Wound extension by means of a graduated ruler, measuring the greatest vertical length (length) and the longest perpendicular length (width). Photographs will be taken with the ruler located on the injury to evaluate the evolution of the wound and the measurement of the perimeter.
3. VU treatment (Annex 4).
4. It will be recorded in the medical record if there has been any concomitant change in your medication since the last visit.
5. It will be recorded in the clinical history if any adverse event or death has occurred since the last visit.

Once the ulcer is closed, a face-to-face visit will be made 15 days after closing to
ensure the same, in which they will perform the same tests and collect the same information described above.

- If the ulcer is healed, a follow-up will be carried out every 15 days of
  security (face-to-face or telephone). This additional follow-up will focus on assessing the long-term safety of DMMCs by collecting adverse events and death events.

All patients should complete the 12-month follow-up period after the last administration.

It is an authentic©copy of an electronic

8. TREATMENT ASSIGNMENT

8.1. RANDOMIZATION

The method of randomization is simple, without restrictions, for it a table of
random numbers. The randomization list will be kept and managed by the Coordination Unit of the promoter, which will be the one who will assign the treatment after confirming compliance with the selection criteria.

8.2. WITHDRAWAL CRITERIA AND PLANNED ANALYSIS OF WITHDRAWALS AND ABANDONMENTS

Inclusion is defined as obtaining the subject's consent to participate in the study, regardless of whether eligibility is confirmed. Patients who have been included will discontinue their participation in the clinical trial if any of the following occur:

- Presence of a serious adverse event since inclusion that, in the opinion of the investigator or sponsor, may jeopardize patient safety or affect the results of the trial.
- Abnormal laboratory value(s), whenever in the opinion of the investigator and/or sponsor the safety of the patient is at risk or may interfere with the interpretation of the results of the study.
- Poor evolution or clinical conditions of the patient that prevent its continuity.
- Indication of prohibited medications or rescue therapies in case of poor wound evolution.
- Technical difficulties in the provision of the investigational drug. In this case, the patient may be re-evaluated when adequate production is possible. The patient will keep his or her assignment number in this case.
- When the patient does not cooperate or does not meet the requirements of the study.
- Withdrawal of consent or loss of follow-up by the patient.
- Pregnancy.

The patient has the right to discontinue the study at any time and any patient may be withdrawn from the study for any reason beneficial to their well-being. If, during the study, the patient's evolution makes it advisable to apply rescue therapies with proven efficacy, the patient will automatically leave the trial, and a failure of the therapy will be recorded in the course of the study will be treated within the guideline that the clinical experts and the health authorities decide.

It is an authentic©copy of an electronic

For the purposes of the safety objective of the trial, safety follow-up of enrolled and withdrawn patients should be ensured throughout the duration of the clinical trial (12 months), to the extent possible. In any case, the date and reason why a subject discontinues participation in the clinical trial should be recorded in the Data Collection Notebook, and the sponsor should be notified immediately if the discontinuation is due to a Serious Adverse Event.

According to good clinical practice, all patients who leave the study prematurely will be given
will attend according to the usual clinical practice. If withdrawal is due to a serious adverse event, patients will be monitored by the investigator or designee until appropriate completion, i.e., until the adverse event disappears or until it is determined to be permanent

8.3. PATIENT REPLACEMENT POLICY

All included patients who have been withdrawn prior to randomization or
once randomized if you have not received any doses of the IEP. Therefore, no patient who withdraws, after receiving the assigned treatment, because of side effects or
ineffectiveness of treatment. For patient replacement, a mirror list will be developed in the randomization system.

8.4. TRIAL INTERRUPTION

The study will be interrupted if any of the following circumstances are met:

1. Serious toxicity (grade 3-4 NCI CTC-AE) related to investigational drug in _3 patients.
2. Serious infections (grade 3-4 NCI CTC-AE) related to the administration procedure in _3 patients.
3. Investigational drug-related mortality in _1 patient

9. DESCRIPTION OF THE TREATMENT

9.1. INVESTIGATIONAL THERAPY

- Investigational product: Adult mesenchymal stem cells of adipose tissue expanded in a biological matrix of fibrin-hyaluronic acid.
    - Fantasy name: BAMS*, Bioengineered Artificial Mesenchimal Sheet.*

It is an authentic©copy of an electronic

- Active substance: Expanded adult trunk mesenchymal cells of adipose tissue.
- Pharmaceutical form: Equivalent of living tissue.
- Administration: Topic.
- Schedule: 4 administrations: one sheet will be administered weekly. Each patient assigned to the experimental treatment group will receive 4 slides at a concentration of about 360,000 cells/cm^2. The BAMS has a surface area of 21cm^2 and contains a total of 7.5 x 10^6 ADSCs.
- Manufacturer of the product under investigation: UPCIT of the Virgen de las Nieves University Hospital.

9.2. COMPARATOR

Conventional treatment (Annex 4).

9.3. PERMITTED AND PROHIBITED CONCOMITANT TREATMENTS

9.3.1. PERMITTED CONCOMITANT TREATMENTS

Jobst Compri2® compression therapy as standard, at 40 mmHg in all cases. Multi-layer compression therapy that provides effective compression for up to 7 days, with the first layer being a padded bandage and the second a cohesive compression bandage.

In case of infection of the ulcer, the administration of oral antibiotics should be allowed to avoid excessive manipulation of the site and the womb.

It is an authentic©copy of an electronic

9.3.2. PROHIBITED CONCOMITANT TREATMENTS

The administration of cytostatic and/or cytotoxic drugs and the typical use of corticosteroids at the site of injury are not permitted.

9.4. INVESTIGATIONAL PRODUCT IDENTIFICATION

The identification and labelling of the investigational product is detailed in Annex IV.

9.5. PROCEDURE FOR ADMINISTERING the IEP

Firstly, exudate will be collected from the wound, to be subsequently studied. After that, the following steps will be carried out:

Cleaning and washing of the wound with normal saline solution, NaCl 0.9% (NaCl 0.9%; BraumTM, 346056); exerting sufficient pressure so that waste or debris is dragged without damaging the wound tissue.

If necessary, mechanical debridement of the devitalized or unrevitalized areas shall be carried out. Healing viability to create an ideal environment for the promotion of the healing process.

Determination of the wound area by means of a graduated ruler, measuring the greatest vertical length (length) and the longest perpendicular length (width), multiplying both lengths.

Application of the sheet of expanded ADSCs in the biological matrix of fibrin on the basis of the wound. Hyaluronic acid. The placement will be carried out in such a way that it covers the entire bed of the ulcer and the periphery. The matrix prepared will be provided by the UPCIT in a simple packaging. The professional in charge of placing the womb will only have to adapt it to the area of the injury.

Wound coverage with a non-adhesive secondary polyurethane foam appliance (New Cutimed® Siltec Plus 73288/001) and Jobst® Compri2® 40mmHg compression therapy.

The day of the first application of treatment will be 0; from that moment on, the
same procedure every 7 days until 4 weeks of treatment is completed. The weekly dose to be received will be a 21cm^2 sheet with 7.5x10^6 ADSCs.

9.6. HEALING PROCEDURE IN THE CONTROL GROUP

Patients assigned to the control group will receive the same treatment, except for the application of the sheet of expanded ADSCs in a biological fibrin-hyaluronic acid matrix. In this case, the non-adhesive polyurethane foam apôsite (New Cutimed® Siltec Plus 73288/001) will not be considered secondary. They will also receive Jobst® Compri2® 40mmHg compression therapy.

It is an authentic©copy of an electronic

10. EVALUATION OF THE RESPONSE

10.1. MAIN VARIABLE

Feasibility

Defined as the possibility of completing the administration of the 4 doses of the investigational drug in at least 80% of patients randomized to the treatment group

Safety:

Incidence of AE, SAE, AR, as well as SUAR, throughout the intervention period, as well as the 12 months after the end of treatment

10.2. SECONDARY VARIABLES

Effectiveness:

The answer will be analysed through clinical and biological parameters

Clinical variables:

- Percentage of patients who achieve complete healing at 12 weeks of follow-up (complete epithelialization maintained for 2 weeks) when comparing control group versus treatment.
- Time elapsed (days) to wound closure, measured from the start of treatment to the absence of continuity solution (when the wound bed is completely re-epithelialized and with new tissue) when comparing control group versus treatment.
- Percentage of patients who presented better in the total score and by Resvech 2.0 scale (size of injury, depth of affected tissues, borders, type of tissue in the wound bed, exudate, and infection · inflammation) with respect to baseline and when comparing control group versus treatment.
- Percentage reduction in wound extension (relative area, measure, graded rule) with respect to baseline visit and when comparing the control group versus the experimental group.
- Percentage of patients who have a decrease in the score of the AVS pain scale with respect to the baseline visit when comparing the control group versus the experimental group.
- Percentage of patients who perceive better quality of life according to the score obtained in the CIVIQ20 throughout the treatment/follow-up with respect to the baseline visit and when comparing both groups.

It is an authentic©copy of an electronic

Biological variables:

- Evolution of the pattern of cytokines and growth factors obtained from ulcer exudate samples at day 0, +7, +14, +21 and +28 when comparing both groups and their relationship with ulcer healing.

11. STATISTICAL ANALYSIS

The statistical analysis will be carried out by an independent biostatistician under the supervision of the project's Principal Investigator. All continuous variables will be described using standardized statistical measurements. The categorical variables will be summarized in frequency tables. For descriptive analyses, absolute or relative frequencies will be used in the case of relative variables. The

quantitative variables, depending on whether they follow a normal distribution (Saphiro-Wilk ($n<50$)), will be shown as m ± SD (mean and standard deviation) and range (minimum and maximum) or P50 [P25-P75]

(median, interquartile range). To compare the different variables in the two groups, the Pearson Chi-square test or the exact Fisher test will be applied in the case of qualitative variables, while , for quantitative variables, the ANOVA and post hoc tests or the Kruskall-Wallis H and Mann-Whitney U tests will be used, always applying the Bonferroni correction (alpha/number of possible comparisons). Values with $p<0.05$ will be considered statistically significant.

12. SAFETY RATING

12.1. ADVERSE EVENTS

All adverse events (AEs) will be recorded in the data collection notebooks from the

moment the patient signs his or her informed consent. As a general rule, the term of diagnosis of the disease will be used. If it is not available, or if the researcher does not consider it

Symptoms or clinical signs can be collected as adverse events until a definitive diagnosis is available.

A deterioration in laboratory or complementary test values, vital signs, or other safety variables, compared to baseline baseline data, should only be reported as an adverse event:

- If it meets any of the criteria to be considered a serious adverse event, or
- If it is the reason for discontinuation of treatment with the investigational product.

For each adverse event recorded in the data collection notebook, the investigator will assess the severity (see section 12.2), the causal relationship (see section 12.3) of these events

It is an authentic©copy of an electronic

with the cell therapy or treatment received (unrelated, unlikely, possible, probable, safe) and/or with any trial procedure and severity (see section 12.3).

12.2. DEFINITIONS

An adverse event (AE) is defined as any adverse medical episode or experience in a patient or clinical trial subject receiving the experimental treatment (including the comparator group), even if it does not necessarily have a causal relationship to that treatment. This may include any unfavorable or unintentional signs or symptoms, an abnormal laboratory finding

(including blood tests, x-rays, or scans), a temporary illness associated with the use of study cell therapy, or a worsening of a pre-existing condition. A disorder

Persistent is a clinical condition (including a disorder being treated) that was diagnosed before the subject signed informed consent and is documented in the subject's medical history. In addition, any event associated with an overdose of the product will also be considered an adverse event.

Medical or surgical procedures (e.g., endoscopy or appendectomy) are not considered an adverse event; The adverse event is the medical condition that gives rise to the procedure.

A procedure-related adverse event is an AE that appears during a study

It is not related to the investigational product, but which, in the opinion of the investigator, is related to some procedure of the trial. For example, a harmful episode that

It is related to a medical procedure required by the protocol such as the surgical conditioning of the patient necessary to administer the investigational drug. The AEs

considered as related to the procedure will be analyzed in isolation, although they will be considered a part of the total related AAs of the study.

Adverse Reaction (AR) An AR is any harmful, unintended reaction to an investigational drug, regardless of the dose administered. Unlike an AE, there is a causal relationship to the investigational drug (possible, probable, or certain relationship).

A Serious Adverse Event (SAE) is defined as any unwanted experience that affects a patient, whether or not it is considered related to the treatment of the protocol and that results in:

- Death.
- A life-threatening event (i.e., there was a risk of immediate death of the patient at the time the reaction was observed).
- Hospitalization or prolongation of a hospitalization.
- Persistent or significant disability/incapacity.

It is an authentic©copy of an electronic

- A congenital anomaly or a birth defect in descendants.
- Any other major medical conditions (i.e., major adverse events that are not immediately life-threatening and do not result in death or hospitalization but may be life-threatening or may require intervention to prevent another of the outcomes listed above, e.g., cancer) according to appropriate medical criteria.
- Suspected transmission of an infectious agent through a drug.

Serious and unexpected adverse reaction (SUAR): A serious adverse reaction whose nature, severity or outcome is not consistent with the baseline safety information.

Toxic death is defined as death secondary to toxicity. This must be specified on the death report form: the cause of death must be listed as toxicity. The assessment of toxic deaths is independent of the assessment of response (patients may die from toxicity after a complete assessment of response to treatment).

Hospitalization is defined as the official admission to a hospital for a period of more than 24 hours. Hospitalization and prolongation of hospitalization are criteria for the severity of an AE; however, they are not considered by themselves a SAE. If there is no AA, the investigator should not report hospitalization or prolongation of hospitalization as a SAE. This is the case in the following situations:

- Hospitalization or prolongation of a hospitalization is needed to perform a procedure required by the protocol.
- Hospitalization or prolongation of a hospitalization is part of a routine procedure of the center (e.g., removal of a vascular stent after surgery).
- Hospitalization due to a pre-existing condition that has not worsened.

All AEs and SAEs suffered by subjects from the signing of the informed consent until the end of their participation in the study should be recorded.

The investigator or his or her collaborator will question and/or examine the patient for signs of adverse events. Questioning of patients regarding the possible occurrence of adverse events should be done in a general way (e.g., “How it has been felt since the last visit?”). The patient should not be questioned about the presence or absence of specific adverse events.

It is an authentic©copy of an electronic

SERIOUS ADVERSE EVENTS MUST BE REPORTED IMMEDIATELY, WITHIN 24 HOURS AFTER THEIR OCCURRENCE TO THE SPONSOR ACCORDING TO THE PROCEDURE DETAILED IN THIS PROTOCOL.

12.3. ASSESSMENT OF THE CAUSALITY AND SEVERITY OF AAS

The investigator will make the causality assessment using the following definitions:

Relationship	Definition
UNRELATED	There is no evidence of any causal relationship.
IMPROBABLE	There is little evidence to suggest a causal relationship (e.g., the event did not occur within a reasonable period of time after administration of study cell therapy). There is another reasonable explanation for the event (e.g. the patient's clinical condition, other concomitant treatments).
POSSIBLE	There is evidence to suggest a possible causal relationship (e.g., because the event occurred within a reasonable time after administration of the study cell therapy). However, the influence of other factors may have contributed to the event (e.g. the patient's clinical condition, other concomitant treatments).
LIKELY	There is evidence to suggest a causal relationship and the influence of other factors is unlikely.
SURE	There is clear evidence to suggest a causal relationship and a possible contribution of other factors can be ruled out.

For causation assessments, events that meet the categories of safe, probable or possibly will be considered "related". Events that are improbably related or unrelated will be considered unrelated. If the investigator believes that a SAE is likely to be unrelated (unlikely related) or unrelated to the study drug, he or she must provide an alternative cause for the study.

The intensity or severity of AEs should be classified according to the common terminological criteria for Events Adverse (CTCAE) Version in effect (

It is an authentic©copy of an electronic

https://ctep.cancer.gov/protocolDevelopment/electronic_applications/ctc.htm). If they are not described, the following definitions may be used to qualify the severity of each AE:

Grade 1	Slight; asymptomatic or mild symptoms, only clinical or diagnostic observations, no intervention indicated.
Grade 2	Moderate; minimal, local or non-invasive intervention; limitation of the instrumental activity of daily life.
Grade 3	Severe; severe or of medical significance but not an immediate threat to the life; hospitalization or prolongation of hospitalization indicated; incapacitating; Limitation in self-care of daily activity
Grade 4	Life-threatening; Urgent intervention required
Grade 5	Death

A distinction must be made between the severity and severity of AEs. An AE that is evaluated as Grade 4 (life-threatening) should not be confused with an SAE. Severity is a category used to rate the intensity of an event; and both an AE and an SAE can be assessed as Grade 4. An event is defined as "serious" when it meets one of the criteria described above in Section 16.2

12.4. PREGNANCIES

Although not considered serious adverse events, if a woman becomes pregnant in the
During a clinical trial, the principal investigator or a collaborator should inform the sponsor and monitor of the clinical trial. In case of pregnancy, it will be followed until its end, as well as the newborn until it reaches one year of age.

12.5. RECORDING AND REPORTING OF ADVERSE EVENTS

12.5.1. NON-SERIOUS ADVERSE EVENTS

All AEs should be recorded and documented in the patient's medical history and in the data collection notebook using standard medical terminology, avoiding the use of ambiguous or colloquial expressions. The dates of commencement and resolution, the severity and the

severity of any adverse event, as well as its relationship to the study drug or procedure. The AE and SAEs will be monitored until they are completely resolved

adding if necessary, a safety period of 30 days once the subject has completed the study, at the discretion of the principal investigator.

It is an authentic©copy of an electronic

The severity, severity and causal relationship of the AE (unrelated, unlikely, possible, probable, certain) shall be assessed in accordance with the guidelines specified in paragraphs 12.2 and 12.3. Measures taken and results obtained (e.g. hospitalization, withdrawal of treatment, etc.) of any adverse events should also be noted. AEs that are likely to be unrelated to the procedure (i.e., reported as unrelated) will not be considered adverse reactions to cell therapy in toxicity analyses and will be reported independently.

12.5.2. SERIOUS ADVERSE EVENTS

If a SAE occurs, whether or not it is related to the study drug and whether or not the investigational product has been administered, the investigator will notify the sponsor within 24 hours of the time the site becomes aware of it by fax, email or email.
exceptionally telephone call through the SAEs notification form. The date and signature of the responsible investigator or one of his or her authorized staff members must appear on
all notification reports. If you have any concerns regarding SAEs, or any concerns regarding the safety of the subject, please contact the sponsor's pharmacovigilance team:

Rosario Mata Alcázar-Caballero / María del Mar Macías Sánchez
Andalusian Network for the Design and Translation of Advanced Therapies - Junta de Andalucía

Serious adverse events that occur at any time after the patient's inclusion in the study should be reported, i.e. from the signing of the informed consent by the
subject and up to 30 days after the patient has completed or left the study. A subject is considered to have completed the study after the last protocol scheduled visit or last contact with the investigator. The date of withdrawal or abandonment will be the date on which the patient and/or investigator determine that the subject can no longer comply with the protocol.

The sponsor shall be responsible for reporting suspected unexpected serious adverse reactions (SUAR) to the investigational medicinal product in accordance with applicable regulations. The reference for determining the expectability of the adverse reaction shall be the baseline safety information contained in the most recent version of the Investigator's Manual. It should be noted that SUARs that have not been previously documented in the Investigator's manual, or that occur

It is an authentic©copy of an electronic

in a more serious form than expected (i.e. they are unexpected), are subject to prompt communication to the Regulatory Authorities by the Promoter.

12.5.3. OVERDOSE

If an overdose is known or suspected to have occurred with the study drug, exceeding the dose indicated in the protocol, whether it is associated with an adverse event, the investigator should inform the sponsor.

If the overdose does not produce clinical sequelae, it will not be considered as a
adverse event. Any clinical sequelae associated with overdose should be considered an adverse event (as described in section 12.5.1) or a serious adverse event (12.5.2). Signs and symptoms, clinical management, and outcome should be reported, if available. An overdose of the investigational product will be considered a SAEA if it is associated with any serious adverse events.

12.6. EXPEDITED NOTIFICATION OF RAGI

The sponsor shall notify the Spanish Agency of Medicines and Health Products of all suspected serious and, at the same time, unexpected adverse reactions associated with the medicinal products in question.
research of which you have been aware, which have occurred in the clinical trial, whether they occur in Spain or in other countries. In all cases, such notification shall be made through the European database Eudravigilance_CTM.

Notification of serious and unexpected adverse reactions by the sponsor to the Agency
The Spanish Association of Medicines and Medical Devices shall in all cases comply with the criteria and procedure specified in Articles 42, 45 and 46 of Regulation (EU) No x536/2014 of the European Parliament and of the Council of 16 April 2014.

Notification will be made in accordance with ICH E2A Definitions and Standards for Expedited Reporting.

13. ETHICAL ASPECTS

13.1. GENERAL CONSIDERATIONS

The sponsor and coordinating investigator of the trial will present to the reference REIC and the Committees of The research of the centers participating in this protocol and all related materials provided to the subject (such as subject information sheets or study descriptions used to obtain informed consent), as well as any compensation provided to the patient. The approval of the aforementioned Committee and the Spanish Agency must be obtained

It is an authentic©copy of an electronic

of the Drug before starting the study and must be documented in a letter to the investigator, specifying the date on which the Investigators met and granting their approval. The investigator must also submit for evaluation by the CEIm and the AEMPS any modifications made to the protocol after receiving approval, in accordance with local legal procedures and requirements.

The trial will be conducted in accordance with the protocol following the sponsor's standard operating procedures and those established at the participating hospital.

The Trial will be conducted in accordance with the recommendations for Clinical Trials and evaluation of A product in the human research phase, which appear in the Declaration of Helsinki, revised in successive world assemblies (WMA, 2013), and the current Spanish Legislation on Clinical Trials (RD 1090/2015). The ICH-GCP (CPMP/ICH/135/95) standards will be followed.

The investigational product meets the definition of "investigational advanced therapy medicine " in Regulation (EC) No 1394/2007 of the European Parliament and of the Council

13.2. STUDY MONITORING

The monitoring will be carried out by personnel belonging to the Coordination Unit of the promoter itself. The responsible monitor will regularly contact and visit the investigator and will be allowed to inspect the various trial registries (CRDs and other relevant data) and source documents, if patient confidentiality is maintained, in accordance with local requirements. The monitor will be responsible for inspecting the CRDs at regular intervals during the study, to Verify compliance with the protocol and that the data included is complete, consistent and accurate. The monitor must have access to lab test reports and other records of patients who are necessary to verify the information included in the CRD. The investigator (or his/her representative) agrees to cooperate with the monitor to ensure that all problems identified in the course of these monitoring visits are resolved. 100% of the data collected will be monitored.

13.3. RETENTION OF STUDY DOCUMENTATION

The Investigator must maintain an adequate and accurate record to allow the study to be fully documented and for the study data to be verified later.

Notification of the termination of the Clinical Trial or premature termination of the Trial will be given in accordance with the requirements described in Article 30 of Royal Decree 1090/2015, of 24 December, which regulates clinical trials with medicines, the Committees for Research Ethics with Medicines and the Spanish Register of Clinical Studies. All documentation related to theclinical rehearsal will be archived in the custody of the Primary Care Unit (UA) of the Granada Metropolitan District , according to current legislation.

It is an authentic©copy of an electronic

13.4. TRIAL INSURANCE

The Andalusian Public Foundation for Progress and Health, as the promoter of the study, has, in accordance with Spanish legislation, civil liability insurance. This policy covers all possible damages that the subject may suffer as a result of the administration of the product under study, in accordance with current legislation (RD1090/2015, article 9).

14. DATA COLLECTION NOTEBOOKS

Investigators, or an authorized representative of the study team, must complete a CRD for each patient enrolled in the study. This also applies to records of patients who do not complete the study. If a patient withdraws from the study, the reason must be indicated in the CRD. If a patient withdraws from the study due to a treatment-limiting adverse event, every effort should be made to clearly document the outcome.

15. FOLLOW-UP AND FINAL REPORTS AND COMMUNICATION OF RESULTS

The principal investigator undertakes to submit the follow-up and final reports, within the deadlines established and communicate, where appropriate, the interruption and the reasons for it. The results of this study will be analyzed and may be published or disseminated in medical journals and/or meetings
scientific. The publications generated will be presented to a committee for the drafting of the study, which will also include the promoters, and the rest of the researchers involved in the study. Since major medical journals restrict, in some cases, the number of authors, the selection of researchers will be based on the recruitment rate and/or tasks performed during the study.

After 12-month follow-up of the patients, the database will be closed and analyzed. Trial data will be published as described above.

16. CONDITIONS FOR PUBLICATION OF RESULTS

The sponsor and the Researchers are responsible for the publication of the results, both positive and negative, of the trial in scientific journals, in accordance with the provisions of article 39 of RD 1090/2015 and based on the uniform requirements for manuscripts submitted to publicationsbiomedical journals (International Committee of Medical Publication Editors, 2016). The publications generated will be presented to a study writing committee, which will include the sponsors and researchers involved in the study.

The results of this study will be analyzed and published or disseminated in medical journals and/or scientific meetings. The publications generated will be presented to a study writing committee, which will include the sponsors and researchers involved in the study.

It is an authentic©copy of an electronic

ANNEX 1: SCHEDULE OF VISITS.

Visits	V 1	V 2	V 3	V 4	V 5	V 6	V 7	V 8	V 9	V 10	V 11	V 12	V 13	V14	V15 - until completion of follow - up 12 months after last infusion
**Identification of the visit**	Selection Randomization	1st Dose Day 0	2nd Dose Day +7	3rd Dose Day +14	4th Dose Day +21	Follow-up visit Day +28	Follow-up visit Day +35	Follow-up visit Day +42	Follow-up visit Day +49	Follow-up visit Day +56	Follow-up visit Day +63	Follow-up visit Day +70	Follow-up visit Day +77	Follow-up visit Day +84	**Post - treatment safety follow - up (every 15 days)**
**Informed Consent**	X														
**Demographic data and lifestyle habits**	X														
**Vital Signs**	X	X	X	X	X	X	X	X	X	X	X	X	X	X	
**Medical and pharmacological history**	X														
**VU rating - Widmer scale**	X														
**Ankle arm index**	X														
**Palpation of tibial pulses**	X	X	X	X	X	X	X	X	X	X	X	X	X	X	
**Hemogram, biochemistry and coagulation**	X					X				X				X	
**Serology**	X														
**Resvech Scale**	X	X	X	X	X	X	X	X	X	X	X	X	X	X	
**MolecuLight i:X Wound Extension**	X	X	X	X	X	X	X	X	X	X	X	X	X	X	

**AVS Scale**	X	X		X		X		X		X		X		X	
**CIVIQ 20 Questionnaire**	X	X			X		X		X		X			X	
**Treatment of VU - BAMS**		X	X	X	X										
**Pregnancy test**	X														
**Sample Collection - Exudate Study**		X	X	X	X	X									
**Adverse events / Mortality**		X	X	X	X	X	X	X	X	X	X	X	X	X	X
**Concomitant medication**	X	X	X	X	X	X	X	X	X	X	X	X	X	X	X

ANNEX 2: STANDARD PROCEDURE FOR ADMINISTRATION OF THE INVESTIGATIONAL MEDICINAL PRODUCT.

1. Remove the compression therapy and then the apôsite that covers the injury. Perform the technique carefully, taking care not to damage the wound bed or the perilesional skin.
2. Sample collection of exudate: with filter paper (FisherbrandTM, Fisher Scientific S.L., Madrid, Spain):
   - Exudate samples should be taken from the ulcer bed by absorption with filter paper (500-1000 ul).
   - Place the filter paper over the injury. It is only necessary to place it, it is not necessary to rub.
   - One minute after the application of the absorbent paper, it shall be placed in 0.5 mL of cold phosphate-buffered saline solution.
   - The samples will be stored at a minimum temperature of -20xC until analysis.
3. Cleaning and washing of the wound with normal saline solution (NaCl 0.9%), exerting sufficient pressure to allow the dragging of debris or debris without damaging the wound tissue.
4. Mechanical debridement of devitalised or non-viable areas of scarring:
   - Gently rub the area of the injury on which you want to perform the technique
   - Wash away non-viable tissue debris
   - Perform a second cleaning of the injury with normal saline solution (NaCl 0.9%), exerting enough pressure to allow the debris or debris to be carried away without damaging the wound tissue.
5. Determine the wound area using a graduated ruler, measuring the longest vertical length (length) and the longest perpendicular length (width), multiplying both lengths.
6. Deposit the fibrin matrix · hyaluronic acid on the base of the wound depending on the size of the injury.
   a) The necessary material for the handling and administration of the matrix is the following:
      - ✓ Sterile cloth
      - ✓ Sterile gloves
      - ✓ Sterile gauze
      - ✓ Normal saline solution (NaCl 0.9%), if necessary

It is an authentic©copy of an electronic

- ✓ Straight blunt tip scissors
- ✓ Simple sterile dissection clamp
- ✓ Static demographic marker (Ref. 400133181)

b) The matrix must remain in cold at all times (2 · 8xC)

c) Remove the mesh from the refrigerator

d) Ensure a sterile field before handling it

e) Keep in mind the area of the injury to calculate the required portion of the matrix

f) Open the container where the mesh is deposited once sterility is guaranteed

g) With the help of the demographic marker and the ruler included in the same package, mark the matrix area you need according to the area of the injury

h) Trim, if necessary, the matrix to fit the wound area

i) Carefully pick up the portion of the matrix with the help of the single-dissecting tweezers

j) Place the piece of womb on the wound without exerting pressure, making sure that it properly covers the injury and that it is in contact with the wound bed

7. Cover the wound with a non-adhesive secondary polyurethane foam appliance (New Cutimed® Siltec Plus 73288/001).

8. Jobst® Compri2® 40mmHg multilayer compression therapy of the area where the injury occurs.

It is an authentic©copy of an electronic

ANNEX 3: STUDY OF EXUDATE

Exudate samples will be stored and analyzed in Laboratory 10.4. Faculty of Health Sciences. University of Granada. Avda de la Ilustraciôn 60. 18071. Granada.

The concentration of IL-4, IL-6, TGFγ1 and IL-10 should be determined in exudate samples collected at the beginning of the study (day 0, visit 2) and at visits 3, 4, 5 and 6. The sample will be collected before cleaning and disinfection of the wound. To this end, samples of at least 500-1000 µl of exudate should be taken from the ulcer bed by absorption with filter paper (FisherbrandTM, Fisher Scientific S.L., Madrid, Spain). Samples will be collected at the same point of the ulcer throughout all visits and will be stored at a minimum temperature of -20xC until analysis after homogenization by pipetting and aliquoting. The samples will be analyzed by ELISA, following the manufacturer's instructions, no later than 6 months after collection and freezing of the exudate. The measurements will be carried out in triplicate. The concentration of each of the cytokines will be expressed as picograms per milliliter (pg/ml). In the event that the concentration of cytokines if exudate is less than the detection limit of the ELISA kit, samples may be concentrated if necessary using centrifugation concentration systems (Millipore).

These samples will only be analysed within the framework of this clinical trial, with the surplus being destroyed according to the centre's protocols.

It is an authentic©copy of an electronic

ANNEX 4: STANDARD PROCEDURE FOR THE CURE OF UNCOMPLICATED VENOUS ULCERS.

1. Remove the compression therapy and then the apôsite that covers the injury. Perform the technique carefully, taking care not to damage the wound bed or the perilesional skin.
2. Cleaning and washing of the wound with normal saline solution (NaCl 0.9%), with sufficient exertion Pressure so that debris or debris can be carried away without damaging wound tissue.
3. Mechanical debridement of devitalised or non-viable areas of scarring:
   - ✓ Gently rub the area of the injury on which you want to perform the technique
   - ✓ Wash away non-viable tissue debris
   - ✓ Perform a second cleaning of the injury with normal saline solution (NaCl 0.9%), exerting enough pressure to allow the debris or debris to be carried away without damaging the wound tissue.
4. Determine the wound area using a graduated ruler, measuring the longest vertical length (length) and the longest perpendicular length (width), multiplying both lengths.
5. Cover the wound with a New Cutimed® Siltec Plus 73288/001Jobst®
6. Traditional compression therapy (Compri2® 40mmHg multilayer)

It is an authentic©copy of an electronic

ANNEX 5: BASIC CEAP CLASSIFICATION

Clinical classification - C

C0: Absence of visible or palpable signs of venous disease C1:

Telangiectasias or reticular veins

C2: Truncal varicose

veins C3: Edema

C4a: Pigmentation and/or eczema

C4b: Lipodermatosclerosis and/or white atrophy

C5: Tissue abnormalities and history of healed venous ulcer C6: Active

venous ulcer

These sections are accompanied by a subheading S or A:

- S: Symptomatic that includes pain, tightness, skin irritation, heaviness, muscle cramps, as well as any other discomfort attributable to venous dysfunction.
- A: Asymptomatic

Etiological classification - E

Ec: Cong§nita

Ep: Primary

Is: Secondary or post-thrombic

In: Unidentified venous etiology Anatomical

classification - A

As: Superficial veins

Ap: Perforating veins

Ad: Veins of the deep venous system An:

Unidentified venous location

Physiopathological classification -

P Pr: Reflux

Po: Obstruction

Pr,o: Reflux and obstruction

Pn: Unidentified venous pathophysiology

It is an authentic©copy of an electronic

ANNEX 6: RESVECH 2.0 SCALE

Items	Measure 0	Measure 1	Measure 2	Measure 3
1. Dimension of the injury				
0. Surface = 0 cm2				
1. Surface < 4 cm2				
2. Surface = 4 ≤ 16 cm2				
3. Surface = 16 ≤ 36 cm2				
4. Surface = 36 ≤ 64 cm2				
5. Surface = 64 ≤ 100 cm2				
6. Surface ≥ 100 cm2				
2. Depth/Tissues Affected				
0. Intact Skin Healed				
1. Dermis-epidermis involvement				
2. Involvement of subcutaneous tissues (adipose tissue without reaching the fascia of the muscle)				
3. Muscle involvement				
4. Involvement of the bone and/or adjoining tissues (tendons, ligaments, joint capsule or black eschar that does not allow you to see the tissues underneath it)				
3. Edges				
0. Indistinguishable				
1. Diffuse				
2. Delimited				
3. Damaged				
4. Thickened				
4. Type of tissue in the wound bed				
4. Necrôtic (dry black eschar or wet)				
3. Necrotic tissue and/or sloughs in the bed				
2. Granulation fabrics				
1. Epithelial tissue				

It is an authentic©copy of an electronic

0. Closure/Healing				
5. Exudate				
3. Dry				
0. Wet				
1. Wet				
2. Saturated				
3. With exudate leakage				
6. Infection/Inflammation (Biofilm signs)				
6.1. Pain that is increasing (Yes=1/No=0)				
6.2. Erythema in the perilesiôn (Si=1/No=0)				
6.3. Edema in the perilesiôn (Si=1/No=0)				
6.4. Temperature increase (Si=1/No=0)				
6.5. Exudate that is increasing (Yes=1/No=0)				
6.6. Purulent exudate (Si=1/No=0)				
6.7. Friable or easily bleeding fabric (Yes=1/No=0)				
6.8. Wound Stagnant what no Progresses (Yes=1/No=0)				
6.9. Biofilm compatible fabric (Si=1/No=0)				
6.10. Odor (Yes=1/No=0)				
6.11. Hypergranulation (Si=1/No=0)				
6.12. Increased wound size (Yes=1/No=0)				
6.13. Satellite lesions (Yes=1/No=0)				
6.14. Tissue pallor (Si=1/No=0)				

It is an authentic©copy of an electronic

ANNEX 7: WIDMER CLASSIFICATION

Wound stage	Clinical signs
I	Edema, subfascial congestion, phlebectasia, varicose veins
II	Induration, pigmentation, eczema
III	Ulcer, ulcerous scar

It is an authentic©copy of an electronic

ANNEX 8: HELSINKI DECLARATION

WORLD MEDICAL ASSOCIATION HELSINKI DECLARATION

Ethical Principles for Medical Research on Human Subjects Adopted by

the

18th World Medical Assembly, Helsinki, Finland, June 1964 and

amended by the

29th World Medical Assembly, Tokyo, Japan, October 1975

35th World Medical Assembly, Venice, Italy, October 1983

41st World Medical Assembly, Hong Kong, September 1989

48th General Assembly Somerset West, South Africa, October

1996 52nd General Assembly, Edinburgh, Scotland, October 2000

Clarification Note, added by the WMA General Assembly, Washington 2002 Clarification Note,

added by the WMA General Assembly, Tokyo 2004

59th General Assembly, Seoul, Korea, October 2008

64th General Assembly, Fortaleza, Brazil, October 2013

Introduction

1. The World Medical Association (WMA) has promulgated the Declaration of Helsinki as a proposal for ethical principles for medical research on human subjects, including research on identifiable human material and information.

The Declaration should be considered as a whole and one paragraph should be applied with consideration of all other relevant paragraphs.

2. In accordance with the mandate of the WMA, the Declaration is intended primarily for physicians. The WMA urges others involved in medical research on human subjects to adopt these principles.

General principles

3. The Geneva Declaration of the World Medical Association links the physician to the formula "to look out only and first and foremost for the health of my patient," and the International Code of Medical Ethics states that: "The physician should consider the best interests of the patient when providing medical care."

4. The physician's duty is to promote and ensure the health, well-being, and rights of patients, including those who participate in medical research. The knowledge and conscience of the physician must be subordinated to the fulfillment of this duty.

It is an authentic©copy of an electronic

5. The progress of medicine is based on research which, in the final analysis, must include studies on human beings.

6. The main purpose of medical research on human beings is to understand the causes, evolution and effects of diseases and to improve preventive, diagnostic and therapeutic interventions (methods, procedures and treatments). Even the best proven interventions must be continually evaluated through research to be safe, efficient, effective, accessible, and of quality.

7. Medical research is subject to ethical norms that serve to promote and ensure respect for all human beings and to protect their health and individual rights.

8. Although the main objective of medical research is to generate new knowledge, this objective should never take precedence over the rights and interests of the person participating in the research.

9. In medical research, it is the duty of the physician to protect life, health, dignity, integrity, the right to self-determination, privacy and confidentiality of the personal information of the people who participate in research. The responsibility for the protection of the people who take part in the research should always lie with a doctor or other health professional and never with the participants in the research, even if they have given their consent.

10. Physicians should consider the ethical, legal, and legal norms and standards for research on human subjects in their own countries, as well as the norms and standards international current. A national or international ethic, legal or legal requirement should not be allowed to diminish or eliminate any measure of protection for persons participating in the investigation set out in this Declaration.

11. Medical research should be conducted in such a way as to minimize potential damage to the environment .

12. Medical research on human beings should be carried out only by people with the education, training and appropriate scientific and ethical qualifications. Research on healthy patients or volunteers needs the supervision of a competent and appropriately qualified physician or other health care professional.

13. Groups that are underrepresented in medical research should have appropriate access to participation in research.

14. The physician who combines medical research with medical care must involve his or her patients in the investigation only to the extent that this demonstrates a justified potential preventive, diagnostic or therapeutic value and if the physician has good reason to believe that the

It is an authentic©copy of an electronic

Participation in the study will not adversely affect the health of patients taking part in the research.

15. Appropriate compensation and treatment should be ensured for people who are harmed during their participation in research.

Risks, Costs and Benefits

16. In the practice of medicine and medical research, most interventions involve some risks and costs.

Medical research on human subjects should only be conducted when the importance of its objective outweighs the risk and costs to the person participating in the research.

17. All medical research on human subjects should be preceded by a careful comparison of the risks and costs to the individuals and groups involved in the research, compared to the foreseeable benefits to them and to other individuals or groups affected by the disease being investigated.

Measures should be implemented to minimize risks. Risks should be continuously monitored, evaluated, and documented by the investigator.

18. Physicians should not engage in human research studies unless they are satisfied that the risks have been adequately assessed and can be met satisfactorily.

When the risks involved are more important than the expected benefits or if they exist
conclusive evidence of definitive results, physicians should evaluate whether to continue, modify, or immediately discontinue the study.

Vulnerable groups and individuals

19. Some groups and individuals under investigation are particularly vulnerable and may be more likely to suffer abuse or further harm.

All vulnerable groups and individuals should receive specific protection.

20. Medical research in a vulnerable group is only justified if the research responds to the health needs or priorities of this group and the research cannot be carried out in a non-vulnerable group. In addition, this group will be able to benefit from the knowledge, practices or interventions derived from the research.

Scientific requirements and research protocols

It is an authentic©copy of an electronic

21. Medical research on human subjects should conform to generally accepted scientific principles and should be supported by a thorough knowledge of the scientific literature, other relevant sources of information, as well as well-conducted laboratory experiments and on animals, where appropriate. Care must also be taken of the welfare of the animals used in the experiments.

22. The project and method of any study on human beings must be clearly described and justified in a research protocol.

The protocol must always refer to the appropriate ethical considerations and must
indicate how the principles set forth in this Declaration have been considered. The protocol should include information on funding, sponsors, institutional affiliations, potential conflicts of interest and incentives for study persons and information on stipulations for treating or compensating persons who have suffered harm as a result of their participation in the research.

In clinical trials, the protocol should also describe appropriate arrangements for post-trial stipulations.

Research Ethics Committees

23. The investigation protocol should be sent for consideration, comment, advice and advice.
approval to the relevant research ethics committee prior to commencing the study. This committee must be transparent in its functioning, it must be independent of the researcher, the
sponsor or any other type of undue influence and must be properly qualified. The committee should consider the laws and regulations in force in the country where the research is conducted, as well as existing international standards, but should not allow these to diminish or eliminate any of the protections for individuals participating in the research set out in this Declaration.

The committee has the right to monitor ongoing trials. The researcher has the obligation to
Provide control information to the Committee, especially on any serious adverse incidents. No amendment should be made to the protocol without the committee's consideration and approval. After the study is complete, the researchers must submit a final report to the committee with a summary of the results and conclusions of the study.

Privacy and confidentiality

24. Every precaution must be taken to protect the privacy of the person participating in the investigation and the confidentiality of his or her personal information.

Informed consent

It is an authentic©copy of an electronic

25. The participation of persons capable of giving informed consent in medical research must be voluntary. Although it may be appropriate to consult family members or community leaders, no person capable of giving informed consent should be included in a study unless he or she freely agrees.

26. In medical research on human beings capable of giving informed consent, each
potential individual should be provided with adequate information about the researcher's objectives, methods, sources of funding, possible conflicts of interest, institutional affiliations, benefits, and benefits.
calculations, foreseeable risks and discomforts arising from the experiment, post-study stipulations, and any other pertinent aspects of the research. The potential person must be informed of the right to participate or not in the research and to withdraw their consent at any time, without exposing themselves to reprisals. Special attention should be paid to the specific information needs of each potential individual, as well as to the methods used to deliver the information.

After making sure that the individual has understood the information, the doctor or other person appropriately qualified must then request, preferably in writing, the informed and voluntary consent of the person. If consent cannot be given in writing, the process for achieving it must be formally documented and witnessed.

All persons participating in medical research should have the option of being informed about the overall results of the study.

27. When requesting informed consent for participation in the research, the physician must
Particular care should be taken when the potential individual is bound to him by a relationship of dependency or if he consents under pressure. In such a situation, informed consent must be requested by a person who is appropriately qualified and has nothing to do with that relationship.

28. When the potential individual is unable to give informed consent, the doctor must seek the informed consent of the legal representative. These people should not be included in the
research that has no potential of benefit to them, unless it aims to promote the health of the group represented by the potential individual and this research cannot be conducted on people capable of giving informed consent and the research involves only minimal risk and cost.

29. If a potential individual participating in the research who is considered incapable of giving informed consent is able to give his or her assent to participate or not in the research, the
The doctor must request it, in addition to the consent of the legal representative. The potential individual's disagreement must be respected.

It is an authentic©copy of an electronic

30. Research on individuals who are physically or mentally unable to give consent, e.g., unconscious patients, can be conducted only if the physical/mental condition that prevents informed consent from being given is a necessary characteristic of the group under investigation. In these circumstances, the doctor must request informed consent from the legal representative. If such a representative is not available and if the research cannot be delayed, the study may be taken to

without informed consent, provided that the specific reasons for including individuals with a disease that does not allow them to give informed consent have been stipulated in the

The research protocol and the study has been approved by a research ethics committee. Consent to continue in the research must be obtained as soon as possible from the individual or a legal representative.

31. The physician must fully inform the patient of the aspects of care that are related to the investigation. The patient's refusal to participate in research or his or her decision to withdraw should never adversely affect the physician-patient relationship.

32. For medical research that uses identifiable human material or data, such as

In the case of research on material or data contained in biobanks or similar deposits, the physician must request informed consent for the collection, storage and reuse. There may be exceptional situations in which it will be impossible or impracticable to obtain consent for such research. In this situation, research can only be carried out after being considered and approved by a research ethics committee.

Use of placebo

33. The potential benefits, risks, costs, and efficacy of any new intervention should be evaluated by comparing it with the best proven interventions, except in the following circumstances:

When there is no proven intervention, the use of a placebo, or no intervention at all, is acceptable; or when for scientifically sound and convincing methodological reasons, it is necessary

To determine the efficacy and safety of an intervention, use any intervention less effective than the best tested, use of a placebo, or no intervention.

Patients who receive any intervention less effective than the best-tested, placebo, or no intervention, will not be at additional risk of serious or irreversible harm as a result of not receiving the best-tested intervention.

Great care must be taken to avoid abusing this option. Post-trial

stipulations

It is an authentic©copy of an electronic

34. Prior to the clinical trial, sponsors, investigators, and host country governments should provide post-trial access to all participants who still need an intervention that has been identified as beneficial in the trial. This information should also be provided to participants during the informed consent process.

Registration and publication of research and dissemination of results

35. All research studies involving human subjects must be entered in a publicly available database before accepting the first person.

36. Researchers, authors, sponsors, directors, and publishers all have strict obligations with respect to the publication and dissemination of the results of his research. Researchers have a duty to make the results of their research on human subjects available to the public and are responsible for the completeness and accuracy of their reports. All parties must agree to the rules § of information delivery. Both negative and inconclusive results and positive results must be published or otherwise made available to the public. The publication should cite the source of funding, institutional affiliations, and conflicts of interest. Research reports that do not adhere to the principles described in this Declaration should not be accepted for publication.

Interventions not tested in clinical practice

37. When in the care of a patient the proven interventions do not exist or other known interventions have proved ineffective, the physician, after seeking expert advice, with the informed consent of the patient or an authorized legal representative, may be permitted to use unproven interventions, if, in his or her opinion, it gives some hope of saving life, restoring health, or alleviating suffering. Such interventions should be further investigated in order to assess their safety and efficacy. In all cases, such new information should be recorded and, where appropriate, made available to the public.

It is an authentic©copy of an electronic
